# No evidence that sociosexual orientation moderates effects of conception probability on women’s preferences for male facial masculinity

**DOI:** 10.1038/s41598-023-37404-6

**Published:** 2023-06-23

**Authors:** Anthony J. Lee, Benedict C. Jones, Brendan P. Zietsch, Patrick Jern, Henry Connolly, Urszula M. Marcinkowska

**Affiliations:** 1 Faculty of Natural Sciences, Division of Psychology, University of Stirling, Stirling, Scotland; 2 School of Psychology, University of Strathclyde, Glasgow, Scotland, UK; 3 Centre for Psychology and Evolution, School of Psychology, University of Queensland, Brisbane, Australia; 4 Department of Psychology, Abo Akademi University, Turku, Finland; 5 Faculty of Health Sciences, Jagiellonian University Medical College, Cracow, Poland

**Keywords:** Human behaviour, Sexual selection

## Abstract

Although many researchers have proposed that women will show stronger preferences for male facial masculinity when conception probability is high, empirical tests of this hypothesis have produced mixed results. One possible explanation for these inconsistent findings is that effects of conception probability on women’s preferences for facial masculinity are moderated by additional factors not typically considered in these empirical tests. One such potential moderator is individual differences in women’s openness to uncommitted sexual relationships (i.e., individual differences in women’s sociosexual orientation); women who are more open to uncommitted sexual relationships might show stronger positive effects of conception probability on masculinity preferences, as their sexuality is more overt and sexual attitudes and behaviours are more diversified. Consequently, we analysed data from three independent samples (*N* = 2304, *N* = 483, and *N* = 339) to assess whether sociosexual orientation moderates the hypothesised positive effect of conception probability on women’s facial masculinity preferences. Analyses showed no evidence that higher conception probability increased preferences for facial masculinity or that sociosexual orientation moderated the effect of conception probability on women’s preferences for facial masculinity. While it remains possible that factors other than sociosexual orientation moderate effects of conception probability on masculinity preferences, our null results suggest that the mixed results for the effects of conception probability on facial masculinity preferences in previous studies are unlikely to be a consequence of failing to consider the moderating role of sociosexual orientation.

## Introduction

It is well established that men with reduced male sex-typical face-shape characteristics (i.e., men with more feminine faces) are attributed pro-social personality characteristics (e.g., trustworthiness^1–3^). Men with exaggerated male sex-typical face-shape characteristics (i.e., men with more masculine faces) have been hypothesized to have stronger immune systems and father healthier offspring^4,5^, although empirical evidence for these associations is weak (for a recent review, see^6^). Because of this putative trade-off between the costs and benefits of choosing a physically masculine partner, many researchers have hypothesized that women will show stronger preferences for men displaying masculine face-shape characteristics at points in the menstrual cycle when conception probability is high and the benefits of choosing a partner with a strong immune system are most likely to be translated into offspring health^4,5^.

Although some early studies reported that women do show stronger preferences for men with masculine face-shapes at points in the menstrual cycle where conception probability is high (e.g., during the ovulatory phase of the menstrual cycle,^5^) these findings have recently been reassessed in light of criticisms of the methodologies that these studies used^6^. One criticism of these early studies is that they have low statistical power due to employing relatively small sample sizes^6,7^, raising the possibility that the positive published findings reflect strong publication bias. Consistent with this possibility, more recent studies that have used (often considerably) larger samples to test whether women show stronger preferences for men with masculine face shapes when conception probability is high have generally reported null results^8–12^.

An alternative explanation for the mixed results for cyclic shifts in women’s preferences for facial masculinity is the existence of individual differences in the extent to which women’s preferences change during the menstrual cycle as a function of their conception probability. Some research has reported positive effects of conception probability on masculinity preferences that occur when women assessed men’s attractiveness for short-term, uncommitted relationships, but not when women assess men’s attractiveness for long-term, committed relationships (e.g.,^5,13^). Null results for effects of conception probability in studies in which no relationship context was specified for which women should assess men’s attractiveness (e.g.,^9,11,12,14^) may be partly a consequence of these context-general attractiveness judgments not reflecting short-term mate preferences. Therefore, the effect of conception probability on masculinity preferences may be more pronounced among women who are more open to uncommitted relationships (i.e., score higher on the Sociosexual Orientation Inventory^13^), particularly when no relationship context is specified for attractiveness judgements. Indeed, previous work has reported that women scoring higher on the Sociosexual Orientation Inventory tend to show stronger preferences for masculine men^14,15^ and that women show stronger preferences for masculine men when instructed to assess men’s attractiveness for a short-term, rather than long-term, relationship^8,16–18^. These latter findings suggest that women who score higher on the sociosexual orientation inventory are more likely to assess men’s attractiveness for short-term relationships.

In light of the above, we analysed three independent datasets (*N* = 2304, *N* = 483, and *N* = 339) to test whether women who reported being more open to uncommitted sexual relationships showed stronger positive effects of conception probability on preferences for masculinized versus feminized versions of men’s faces. If sociosexual orientation did moderate the relationship between conception probability and facial masculinity preferences, we would expect to see a significant positive interaction between the effects of conception probability and sociosexual orientation on masculinity preferences.

## Methods

### Participants

Data were collected from three independent samples. All participants completed the studies online.

#### Sample 1

Sample 1 is from a large, cross-national survey investigating human mating^19,20^. Participants were online volunteers recruited via social media, university platforms, and personal communication. A total of 8957 women completed the online survey. Participants who did not meet the inclusion criteria were removed from the sample; this included not being exclusively heterosexual (*N* = 1958), currently pregnant (*N* = 261), currently breast feeding (*N* = 429), reporting having an irregular menstrual cycle length (*N* = 1871), currently using hormonal contraception (*N* = 1499), missing data on key variables (*N* = 552), or residing in a country where less than 10 participants were recruited from that country (following Lee, DeBruine and Jones^21^, N = 83). This resulted in a final sample of 2304 women (*M* = 26.85 years, *SD* = 7.62 years) from 25 countries (Australia, Brazil, China, Colombia, Croatia, Estonia, Finland, France, Iran, Italy, Latvia, Mexico, Nepal, New Zealand, Nigeria, Poland, Romania, Russia, Saudi Arabia, Singapore, Slovakia, Spain, Sweden, United Kingdom, and United States). For more information on this sample, see Marcinkowska et al.^20^ and Marcinkowska et al.^19^.

#### Sample 2

Participants were a subsample from the Genetics of Sexuality and Aggression Twin (GSAT) sample^22^ and were 2166 women who were either identical or nonidentical twins or their female siblings. Participants were removed from the sample if they were not exclusively heterosexual (*N* = 248), currently pregnant (*N* = 122), currently breast feeding (*N* = 149), reported having an irregular menstrual cycle (*N* = 154), currently using hormonal contraception (*N* = 633), or missing data on key variables (*N* = 377). This resulted in a final sample of 483 women (*M* = 34.31 years, *SD* = 5.31 years). For more information on this sample, see Zietsch, Lee, Sherlock and Jern^23^ and Johansson et al.^22^.

#### Sample 3

Participants were online volunteers recruited via social media or undergraduate students at the University of Stirling. A total of 1507 participants completed the survey; however, participants were removed if they reported not being female (*N* = 44), not exclusively being heterosexual (*N* = 17), currently pregnant or breastfeeding (*N* = 101), reported having an irregular menstrual cycle (*N* = 347), currently using hormonal contraception (*N* = 238), or missing data on key variables (*N* = 420). This resulted in a final sample of 339 women (*M* = 25.35 years, *SD* = 7.57 years).

### Sociosexual orientation

For Sample 1 and 3, sociosexual orientation was measured using the revised sociosexual orientation inventory^13^. This questionnaire measures participant’s orientation towards uncommitted sex in three domains: past behavioural experiences, attitudes towards uncommitted sex, and desire for sex. Each item is scored on a 9-point scale, with a total sociosexual orientation inventory (SOI) score being calculated as the sum of all items. Higher scores on this measure indicates a more unrestricted sociosexual orientation (i.e., greater openness to uncommitted sexual relationships).

For Sample 2, sociosexual orientation was measured using the original sociosexual orientation inventory^24^. As in Zietsch et al.^23^, scores for each item were standardised, and outliers were winsorised (± 3 *SD*). Participant SOI was calculated as the mean of these standardised, winsorised scores, with higher scores indicating a more unrestricted sociosexual orientation.

### Conception probability

For all three samples, conception probability was estimated based on several items regarding menstrual cycle. These included the start date of the most recent menstrual cycle, the average or normal number of days between menses (i.e., menstrual cycle length), and the extent to which the menstrual cycle fluctuates from month to month.

We used four methods reported in previous literature to calculate conception probability scores from self-reported data. The four methods differ in (1) whether cycle day is calculated using the count-forward or count-back method, and (2) whether conception probability is a dichotomous or continuous variable. The count-forward method involved calculating estimated cycle day by counting the number of days between the date women reported as the start of the last menses and the date they completed the questionnaire. The count-back method involved calculating estimated cycle day by counting the number of days between the date they completed the questionnaire and the predicted date of their next menses. Following Penton-Voak et al.^5^, for dichotomous conception probability, high conception probability was operationalised as being between day 6 and day 14 of their menstrual cycle (i.e., the follicular phase), while all other times were considered low conception probability (i.e., days 0 to 5 and 15 till the last day of the cycle). Continuous conception probability percentage was estimated from the cycle day following methods described in Wilcox, Dunson, Weinberg, Trussell and Baird^25^, which provides a conversion table of likelihood of conception according to cycle day given a single act of sexual intercourse.

### Facial masculinity preference

For all three samples, preferences for facial masculinity were measured using a two-alternative forced-choice task. This is where participants are presented with two identical faces that have been subtly manipulated on the facial masculinity-femininity dimension, of which they then reported which face they found more attractive. For all samples, stimuli manipulation was done following established procedures using the Psychomorph program^26^. This involved calculating the linear difference between a composite male face and a composite female face. Facial masculinity was manipulated by adding or subtracting 50% of this difference to each individual face, producing a masculinised and feminised version of each face. For more information on the manipulation procedure, see Perrett et al.^3^. For all three samples, for each participant the order of faces and whether the masculinised face was presented on the left or right side was randomised.

For Sample 1, participants were shown 20 pairs of male faces (aged 18–24 years), each with a neutral expression. Participants were asked to select which face they found more attractive. For more details on visual stimuli in this dataset, see Marcinkowska, Kozlov, Cai, Contreas-Garduño, Dixson, Oana et al.^27^.

For Sample 2, participants were shown 21 pairs of male faces (aged 19–31 years) with neutral expressions from the FACES database^28^. Participants were asked to rate which face they found more attractive on an 8-point scale (1 = left is much more attractive, 8 = right is much more attractive). Responses were coded such that higher numbers indicate a greater preference for facial masculinity. See Zietsch et al.^23^ for more detail.

For Sample 3, participants were shown 42 pairs of male faces sourced from the 3D.sk image set^29^. Similar to Sample 1, participants were simply asked to choose the face they found more attractive.

### Statistical analysis

For all three samples, data was analysed using mixed effects modelling. Binomial mixed effects models were used for Sample 1 and 3, while linear mixed effects models were used for Sample 2. Analyses were conducted in the R statistical software^30^ using the *lme4*^31^ and *lmerTest* packages^32^. Across all samples, the outcome variable was preference for facial masculinity, while predictors included participants’ SOI score, conception probability, and their interaction term. Predictors were either z-standardised (for continuous variables), or effect coded (for dichotomous variables) before being entered in the model. Random intercepts were specified for grouping factors of Participant ID and Stimulus ID, following best practice^33^. Additional grouping factors of country of residence and world region, and family were included for Sample 1 and Sample 2 respectively to account for non-independence in the data. Random slopes were specified maximally according to Barr, Levy, Scheepers and Tily^34^ and Barr^35^.

Separate models were conducted for each method of calculating conception probability. Here, we report the estimated fixed effects for the model where conception probability was calculated using the continuous, count-forward method. For full results for all models (including analysis code) see the supplementary materials or on the OSF (https://osf.io/ch53f/), though the pattern of results remains unchanged regardless of the method used (except where noted). Also, given the homogeneity in the designs across the three studies, we pooled the three samples and analysed the data using a single binomial mixed effects model—these analyses are reported in the supplementary materials and on the OSF (https://osf.io/ch53f/).

### Ethics statement

The study was conducted in compliance with national legislation and the Code of Ethical Principles for Medical Research Involving Human Subjects of the World Medical Association (Declaration of Helsinki). Informed consent was obtained from all participants. For Sample 1, ethics approval was given by the Jagiellonian University Medical College Ethics Board. For Sample 2, ethics approval was given by The Ethics Committee of the Abo Akademi University (Turku, Finland). For Sample 3, ethics approval was given by the University of Stirling General University Ethics Panel.

## Results

### Sample 1

The estimated fixed effects for the continuous, count-forward model with Sample 1 are reported in Table 1. There was a significant main effect of SOI, such that more unrestricted participants had a greater preference for facial masculinity. There was no significant main effect of conception probability, nor was the interaction term significant. All other models produced the same patterns of results, except for the continuous, count-back model, where the main effect of SOI was non-significant.

**Table 1:** The estimated fixed effects from Sample 1 for the model where conception probability was calculated using the continuous, count-forward method.

	Estimate (Std. error)	*z*-value (d)	*p*-value
Intercept	.14 (.30)	.46	.648
SOI	.15 (.05)	2.74	.006**
Conception probability	.01 (.08)	.18	.857
SOI * conception probability	− .03 (.04)	− .75	.456

*p < .05, **p < .01, ***p. < .001.

### Sample 2

The estimated fixed effects for the continuous, count-forward model with Sample 2 are reported in Table 2. There was no significant main effect of SOI or conception probability, nor was the interaction term significant.

**Table 2:** The estimated fixed effects from Sample 2 for the model where conception probability was calculated using the continuous, count-forward method.

	Estimate (Std. Error)	*t*-value (approx. df)	*p*-value
Intercept	5.25 (.14)	37.72 (22.95)	< .001 ***
SOI	.05 (.04)	1.07 (43.14)	.291
Conception probability	− .08 (.04)	− 1.92 (195.90)	.057
SOI * conception probability	.03 (.05)	.56 (121.78)	.579

*p < .05, **p < .01, ***p. < .001.

### Sample 3

The estimated fixed effects for the continuous, count-forward model with Sample 3 are reported in Table 3. Similar to Sample 2, there was no significant main effect of SOI or conception probability, nor was there a significant interaction.

**Table 3:** The estimated fixed effects from Sample 3 for the model where conception probability was calculated using the continuous, count-forward method.

	Estimate (Std. Error)	*z*-value	*p*-value
Intercept	.67 (.14)	4.76	< .001 ***
SOI	.08 (.04)	1.86	.063
Conception probability	.02 (.05)	.49	.628
SOI * conception probability	.01 (.05)	.22	.825

*p < .05, **p < .01, ***p. < .001.

### Aggregated analyses

At the request of a reviewer, we re-analysed the data with the outcome variable as the aggregated masculinity preference calculated across stimuli. These analyses showed the same pattern of results as those reported above (i.e., there was no significant interaction between SOI and conception probability), with one exception. In sample 1, for the count-forward continuous model, there was a significant interaction between SOI and conception probability. However, this interaction was in the opposite direction to predictions (i.e., women with a more restricted sociosexual orientation showed an increased preference for facial masculinity when conception probability is high). As such, our conclusion does not differ depending on the analytic approach. Full results are reported in the supplementary materials.

## Discussion

The current study analysed three different datasets, testing for interactions between the effects of conception probability and sociosexual orientation on woman’s preferences for male facial masculinity. None of these analyses showed a significant interaction between conception probability and sociosexual orientation (i.e., none of our analyses showed evidence that sociosexual orientation moderated the effect of conception probability on women’s preferences for male facial masculinity). Collectively, these null results suggest that inconsistent results for the effects of conception probability on masculinity preferences in previous studies are unlikely to be a consequence of those studies not having considered the moderating role of individual differences in women’s sociosexual orientation. Of course, our research does not speak to the question of whether other individual-difference measures and external factors moderate this putative relationship. Further research would be needed to clarify that issue.

Some researchers have hypothesised that women who are more open to uncommitted sexual relationships will show stronger preferences for masculine characteristics in men’s faces (e.g.,^15,36^). Support for a significant main effect of sociosexual orientation on women’s preferences for facial masculinity was inconsistent across our three samples. This inconsistency could be due to methodological differences between studies. For example, the effects of sociosexual orientation on facial masculinity preferences may be specific depending on stimulus sets. Alternatively, these inconsistencies may be due to the different versions of the sociosexual orientation inventory being used across samples or differences in the ages of the samples tested (the mean age for Sample 2 was 34.31 years, compared to 26.85 years and 25.35 years of Samples 1 and 3 respectively). We note, however, that the estimate of the effect of sociosexual orientation on women’s facial masculinity preferences was positive in all three samples. Therefore, our studies may support the existence of a positive, albeit weak, association between sociosexual orientation and masculinity preferences.

A strength of our study is that we have analysed data from three diverse and heterogenous samples. In addition, the three samples used different stimulus sets. As such, the consistency of our null results for the interaction between conception probability and sociosexual orientation on facial masculinity preferences suggests these null findings would likely generalise well to new samples. However, there are also several potentially important limitations to note.

First, we employed a cross-sectional design that is not ideal for detecting effects of conception probability on women’s masculinity preferences. While the large sample size in our combined analysis goes some way to offsetting this limitation, further work using a more powerful longitudinal design to test for an effect of conception probability may yet reveal a moderating effect of sociosexual orientation because such designs better control for unmeasured individual differences in face preferences.

Second, we used a forced choice paradigm to assess women’s preferences for masculinised versus feminised male faces, rather than a rating paradigm in which individual faces were rated for attractiveness. The forced choice approach may be suboptimal, given studies showing that these two testing paradigms can produce qualitatively different results^37–39^. While some empirical work has suggested that masculinity preferences assessed using the forced choice paradigm are a better predictor of the masculinity of women’s actual and ideal partners than are masculinity preferences assessed using a rating paradigm^40^, further research using paradigms in which individual faces are rated for attractiveness might also reveal changes in masculinity preferences that are not apparent using the forced choice testing method.

In conclusion, we found little evidence that sociosexual orientation moderates an effect of conception probability on women’s preferences for male facial masculinity. While our null results do not speak to the possibility that other factors moderate conception-probability effects, and notwithstanding the methodological limitations described above, these null results suggest that not accounting for individual differences in sociosexual orientation in previous studies is unlikely to explain inconsistent results for the hypothesised link between conception probability and women’s facial masculinity preferences.

## Supplementary Information

Supplementary Information 1.

Supplementary Information 2.

Supplementary Information 3.

Supplementary Information 4.

Supplementary Information 5.

## Data Availability

The datasets generated and analysed during the current study are available on the OSF repository at https://osf.io/ch53f/.
