# Supplementary material for: No evidence that sociosexual orientation moderates effects of conception probability on women’s preferences for male facial masculinity
Source: Sci Rep. 2023 Jun 23;13:10245. doi: 10.1038/s41598-023-37404-6 (PMC10290078; doi:10.1038/s41598-023-37404-6)
Supplement: Supplementary file 1 — Supplementary Information 1. [file 41598_2023_37404_MOESM1_ESM.docx]

*Aggregated Analyses*

At the request of a reviewer, we re-analysed the data where the outcome variable is the aggregated masculinity preferences across the multiple face-pair trials. For Samples 1 and 3, scores on this aggregated measure ranged from 0 (where the feminine face was chosen for all trials) to 1 (where the masculine face was chosen for all trials). For Sample 2, the aggregated score was the mean rating given by a participant across all trials, where higher scores indicate a greater preference for facial masculinity. For analyses involving the continuous conception probability score, the data was analysed using a linear regression, with predictors being SOI, conception probability, and the interaction between the two. For analyses involving the dichotomous conception probability score, data was analysed via a between-subjects ANOVA, with SOI, conception probability, and the interaction between the two entered as IVs.

For all analyses conducted for Samples 2 and 3, there was no change in the pattern of results; there was no significant main effect of SOI or conception probability on facial masculinity preferences, and there was no significant interaction. Analysis code and full results for all analyses are available on the OSF (<https://osf.io/ch53f/>).

For Sample 1, across all analyses, we replicated the main effect of SOI from the mixed effects model where participants who reported a more unrestricted sociosexual orientation reported a greater preference for facial sexual dimorphism. However, unlike with the mixed effects model, analyses with the continuous conception probability measure found a significant interaction between SOI and conception probability (as an example, results for the continuous count-forward model are reported below in Table S2). However, the interaction term is negative, which would suggest that women with a more restricted sociosexual orientation show an increase in facial masculinity preferences when conception probability is high. This is in the opposite direction to what we predicted.

Table S2. The estimated regression coefficients where conception probability was calculated using the continuous, count-forward method.

|  | Std. Estimate | *t*-value (df = 2272) | *p*-value |
| --- | --- | --- | --- |
| SOI | .21 | 10.01 | < .001*** |
| Conception Probability | .06 | 2.69 | .007** |
| SOI * Conception Probability | -.07 | -3.66 | < .001*** |

* *p* < .05, ** *p* < .01, *** *p. <* .001

Given that a significant interaction was found in only some of the analyses conducted using Sample 1 (and the interaction is in the opposite direction to predictions), that no significant interactions were found for Samples 2 and 3, and that the interaction was non-significant for all analyses using mixed effects modelling, overall the results do not support the notion that the effects of conception probability on masculinity preferences is moderated by women’s sociosexual orientation.
