# Supplementary material for: No evidence that sociosexual orientation moderates effects of conception probability on women’s preferences for male facial masculinity
Source: Sci Rep. 2023 Jun 23;13:10245. doi: 10.1038/s41598-023-37404-6 (PMC10290078; doi:10.1038/s41598-023-37404-6)
Supplement: Supplementary file 2 — Supplementary Information 2. [file 41598_2023_37404_MOESM2_ESM.docx]

*Combined Analysis*

Given the homogeneity in the designs across the three studies, we combined the three samples and analysed the data is a single binomial mixed effects model. For this analysis, predictors were mean-centred within studies, and the model included an additional random effects grouping factor of sample. For Sample 2, ratings were dichotomised to match that of the other samples; scores of 4 or below (indicating a preference for the feminised face) were coded as 0, while scores of 5 and above (indicating a preference for the masculinised face) were coded as 1.

The estimated fixed effects from the combined model are reported in Table 4. There was a significant main effect of SOI, such that those who are more unrestricted had a greater preference for facial masculinity. There was no significant main effect of conception probability, nor was the interaction term significant. All other models where conception probability was calculated using different methods produced the same pattern of results. We note, however, that results of these combined analyses will predominantly be driven by patterns in the largest dataset.

Table S1. The estimated fixed effects for the combined model where conception probability was calculated using the continuous, count-forward method.

|  | Estimate (Std. Error) | *z*-value | *p*-value |
| --- | --- | --- | --- |
| Intercept | .65 (.24) | 2.69 | .007 ** |
| SOI | .17 (.06) | 2.62 | .009** |
| Conception Probability | .00 (.05) | .04 | .969 |
| SOI * Conception Probability | -.02 (.05) | -.52 | .606 |

* *p* < .05, ** *p* < .01, *** *p. <* .001
