# Supplementary material for: No evidence that sociosexual orientation moderates effects of conception probability on women’s preferences for male facial masculinity
Source: Sci Rep. 2023 Jun 23;13:10245. doi: 10.1038/s41598-023-37404-6 (PMC10290078; doi:10.1038/s41598-023-37404-6)
Supplement: Supplementary file 3 — Supplementary Information 3. [file 41598_2023_37404_MOESM3_ESM.pdf]

Conception Probability and Sociosexual Orientation on Facial Masculinity Preferences - Sample 1

Contents

Load Packages and Custom Functions **1**

Load Data **2**

Participant Demographics **3**

Prepare Data **3**

Analyses - Does SOI interact with conception risk to predict facial masculinity preference. **4**

Count-forward - Continuous 4

Count-Forward Dichotomous 7

Count-Back - Continuous 9

Count-Back Dichotomous 11

Load Packages and Custom Functions

```
library(tidyverse)
library(lme4)
library(lmerTest)
library(gghalves)

masc2 <- function(x){
    out <- recode(x,`1` = 0,`2` = 1)
    return(out)
}

masc1 <- function(x){
    out <- recode(x,`1` = 1,`2` = 0)
    return(out)
}

checkN <- function(data,label){
  print(paste(label,": ",NROW(data),sep = ""))
  return(data)
}

z <- function(x,remove.outliers = FALSE,winsorise = FALSE){
    out <- (x - mean(x,na.rm = TRUE))/sd(x,na.rm = TRUE)
    if (remove.outliers == TRUE){
      out <- ifelse(out >3,NA,ifelse(out < -3,NA,out))
    }
    if (winsorise == TRUE){
```

```
      out <- ifelse(out > 3,3,ifelse(out < -3,-3,out))
    }
    return(out)
}
```

Load Data

```
data <- read.csv("masculinity.data.csv",stringsAsFactors = FALSE) %>%
    mutate(
    m1 = masc2(m1),
    m2 = masc2(m2),
    m3 = masc2(m3),
    m4 = masc2(m4),
    m5 = masc1(m5),
    m6 = masc1(m6),
    m7 = masc1(m7),
    m8 = masc1(m8),
    m9 = masc2(m9),
    m10 = masc2(m10),
    m11 = masc2(m11),
    m12 = masc2(m12),
    m13 = masc1(m13),
    m14 = masc1(m14),
    m15 = masc1(m15),
    m16 = masc1(m16),
    m17 = masc2(m17),
    m18 = masc2(m18),
    m19 = masc2(m19),
    m20 = masc2(m20),
    f1 = masc2(f1),
    f2 = masc1(f2),
    f3 = masc2(f3),
    f4 = masc1(f4),
    f5 = masc2(f5),
    f6 = masc2(f6),
    f7 = masc2(f7),
    f8 = masc2(f8),
    f9 = masc2(f9),
    f10 = masc1(f10),
    f11 = masc2(f11),
    f12 = masc2(f12),
    f13 = masc2(f13),
    f14 = masc1(f14),
    f15 = masc2(f15),
    f16 = masc1(f16),
    f17 = masc1(f17),
    f18 = masc1(f18),
    f19 = masc2(f19),
    f20 = masc2(f20)
    )

participant.data <- read.csv("participant.data.csv",stringsAsFactors = FALSE)
```

Participant Demographics

```
#Number of men and women in full sample
table(participant.data$sex)
```

```
##
## Female   Male
##   8957   3473
```

```
#Total number of countries in full sample
NROW(unique(participant.data$country))
```

```
## [1] 116
```

```
#Mean and SD for age in full sample
summarise(participant.data, mean.age = mean(age,na.rm = TRUE),
                sd.age = sd(age,na.rm = TRUE))
```

```
##   mean.age   sd.age
## 1   27.624 9.172805
```

Prepare Data

```
participant.data <- participant.data %>% checkN("Full Sample") %>%
  filter(sex == "Female") %>% checkN("Female") %>%
  mutate(soi = soi1 + soi2 + soi3 + soi4 + soi5 + ((soi6*-1) + 10) + soi7 + soi8 + soi9) %>%
    filter(sexo == 1) %>% checkN("Exclusively Heterosexual") %>%
  filter(pregnant == "No") %>% checkN("Not Pregnant") %>%
  filter(lactating == "No") %>% checkN("Not Lactating") %>%
  filter(menstraul.regular == "Yes") %>% checkN("Regular Cycle") %>%
  filter(hormonal.contraception == "No") %>% checkN("Not Using Hormonal Contraception") %>%
  mutate(menstrual.last = ifelse(!is.na(menstrual.last_recode),menstrual.last_recode,menstrual.last),
         menstrual.last = as.numeric(menstrual.last)) %>%
  filter(!is.na(menstrual.last)) %>% checkN("Missing Menstrual Last") %>%
  filter(menstrual.length <= 38 & menstrual.last <= 38) %>% checkN("Menstrual Length Out of Bounds") %>
  filter(!is.na(soi)) %>% checkN("Missing SOI") %>%
  filter(!is.na(country)) %>%
    group_by(country) %>%
    filter(n() >= 10) %>% checkN("More than 10 participants per country") %>%
  ungroup() %>%
  left_join(read.csv("conception.risk.csv",stringsAsFactors = FALSE),by = c("menstrual.last" = "day")) %
  select(ResponseId,country,sex,age,menstrual.length,menstrual.last,soi,cr_cf.cont = conception.risk) %>
  mutate(cr_cf.dich = ifelse(menstrual.last >= 6 & menstrual.last <= 14,"High","Low")) %>%
  mutate(count.back = 28 - (menstrual.length - menstrual.last),
         count.back = ifelse(count.back < 0,NA,count.back),
         cr_cb.dich = ifelse(count.back >=6 & count.back <= 14,"High","Low"),
         cr_cb.dich = ifelse(is.na(count.back),NA,cr_cb.dich)) %>%
  left_join(read.csv("conception.risk.csv",stringsAsFactors = FALSE),by = c("count.back" = "day")) %>%
  select(ResponseId,country,sex,age,soi,cr_cf.cont:cr_cb.dich,cr_cb.cont = conception.risk,-count.back)
  mutate(z.soi = c(scale(soi)),
         z.cr_cf.cont = c(scale(cr_cf.cont)),
         e.cr_cf.dich = recode(cr_cf.dich,"High" = .5,"Low" = -.5),
         z.cr_cb.cont = c(scale(cr_cb.cont)),
         e.cr_cb.dich = recode(cr_cb.dich,"High" = .5,"Low" = -.5))
```

```
## [1] "Full Sample: 13313"
## [1] "Female: 8957"
## [1] "Exclusively Heterosexual: 6999"
## [1] "Not Pregnant: 6738"
## [1] "Not Lactating: 6309"
## [1] "Regular Cycle: 4438"
## [1] "Not Using Hormonal Contraception: 2939"
## [1] "Missing Menstrual Last: 2846"
## [1] "Menstrual Length Out of Bounds: 2819"
## [1] "Missing SOI: 2387"
## [1] "More than 10 participants per country: 2304"
```

```r
#Number of men and women in full sample
table(participant.data$sex)
```

```
##
## Female
##   2304
```

```r
#Total number of countries in full sample
NROW(unique(participant.data$country))
```

```
## [1] 25
```

```r
sort(unique(participant.data$country)) %>% paste(collapse = ", ")
```

```
## [1] "Australia, Brazil, China, Colombia, Croatia, Estonia, Finland, France, Iran, Islamic Republic o
```

```r
#Mean and SD for age in full sample
summarise(participant.data, mean.age = mean(age,na.rm = TRUE),
                sd.age = sd(age,na.rm = TRUE))
```

```
## # A tibble: 1 x 2
##   mean.age sd.age
##      <dbl>  <dbl>
## 1     26.8   7.62
```

```r
rating.data <- select(data,ResponseId,m1:f20) %>%
    filter(ResponseId %in% participant.data$ResponseId) %>%
    gather(key = "faceId",value = "response",m1:f20)

country.data <- read.csv("Updated Country.Data.csv",stringsAsFactors = FALSE) %>%
  select(Country,region)

analysis.data <- left_join(participant.data,rating.data,by = "ResponseId") %>%
    left_join(country.data,by = c("country" = "Country"))

write.csv(analysis.data,"analysis.data_sample1.csv",row.names = FALSE)
```

Analyses - Does SOI interact with conception risk to predict facial masculinity preference.

Count-forward - Continuous

Linear Mixed Effects Model

```r
if(file.exists("model.cf_cont.Rdata")){
  load("model.cf_cont.Rdata")
} else {
  model.cf_cont <- glmer(response ~ z.soi*z.cr_cf.cont +
                    (1 | ResponseId) +
                    (1 + z.soi*z.cr_cf.cont || faceId) +
                    (1 + z.soi*z.cr_cf.cont || country) +
                    (1 + z.soi*z.cr_cf.cont || region),data = analysis.data,family = "binomial")
  save(model.cf_cont,file = "model.cf_cont.Rdata")
}

summary(model.cf_cont)
```

```
## Generalized linear mixed model fit by maximum likelihood (Laplace
##   Approximation) [glmerMod]
##  Family: binomial  ( logit )
## Formula: response ~ z.soi * z.cr_cf.cont + (1 | ResponseId) + (1 + z.soi *
##     z.cr_cf.cont || faceId) + (1 + z.soi * z.cr_cf.cont || country) +
##     (1 + z.soi * z.cr_cf.cont || region)
##    Data: analysis.data
##
##      AIC      BIC   logLik deviance df.resid
##  50289.9  50438.2 -25127.9  50255.9    45504
##
## Scaled residuals:
##     Min      1Q  Median      3Q     Max
## -7.8394 -0.6564  0.1962  0.6471  6.4732
##
## Random effects:
##  Groups     Name               Variance  Std.Dev.
##  ResponseId (Intercept)        1.510e+00 1.2286372
##  country    z.soi:z.cr_cf.cont 8.006e-08 0.0002829
##  country.1  z.cr_cf.cont       9.106e-05 0.0095424
##  country.2  z.soi              9.414e-07 0.0009703
##  country.3  (Intercept)        7.295e-02 0.2700939
##  faceId     z.soi:z.cr_cf.cont 8.515e-03 0.0922757
##  faceId.1   z.cr_cf.cont       7.936e-03 0.0890818
##  faceId.2   z.soi              3.214e-02 0.1792676
##  faceId.3   (Intercept)        1.041e+00 1.0201350
##  region     z.soi:z.cr_cf.cont 2.353e-07 0.0004851
##  region.1   z.cr_cf.cont       4.328e-04 0.0208032
##  region.2   z.soi              6.126e-06 0.0024752
##  region.3   (Intercept)        1.981e-01 0.4451095
## Number of obs: 45521, groups:
## ResponseId, 2276; country, 25; faceId, 21; region, 7
##
## Fixed effects:
##                    Estimate Std. Error z value Pr(>|z|)
## (Intercept)         0.13741    0.30112   0.456  0.64816
## z.soi               0.14770    0.05388   2.741  0.00612 **
## z.cr_cf.cont        0.01449    0.08022   0.181  0.85668
## z.soi:z.cr_cf.cont -0.02905    0.03900  -0.745  0.45646
```

```
## ---
## Signif. codes:  0 '***' 0.001 '**' 0.01 '*' 0.05 '.' 0.1 ' ' 1
##
## Correlation of Fixed Effects:
##              (Intr) z.soi  z.cr_.
## z.soi         0.012
## z.cr_cf.cnt -0.005 -0.031
## z.s:z.cr_c. -0.017 -0.095  0.196
## optimizer (Nelder_Mead) convergence code: 0 (OK)
## Model failed to converge with max|grad| = 0.025885 (tol = 0.002, component 1)
```

Plot Data

```
plot.data <- analysis.data %>%
  mutate(soi_group = factor(ifelse(soi >= median(soi),"More Unrestricted","More Restricted"),levels = c
    group_by(ResponseId,cr_cf.cont,soi_group) %>%
    summarise(
                mean.response = mean(response,na.rm = TRUE),
                se.response = sd(response,na.rm = TRUE)/n()
                ) %>%
    filter(!is.na(mean.response))

ggplot(plot.data,aes(x = cr_cf.cont,y = mean.response,group = soi_group,colour = soi_group)) +
    geom_pointrange(aes(ymin = mean.response - se.response,ymax = mean.response + se.response),size = .
    geom_smooth(data = plot.data,method = "lm") +
    theme_classic() +
    xlab("Conception Risk (Count-Forward Continuous)") +
    ylab("Mean Masculinity Preference (with SE)") +
  labs(colour = "Sociosexual Orientation")
```

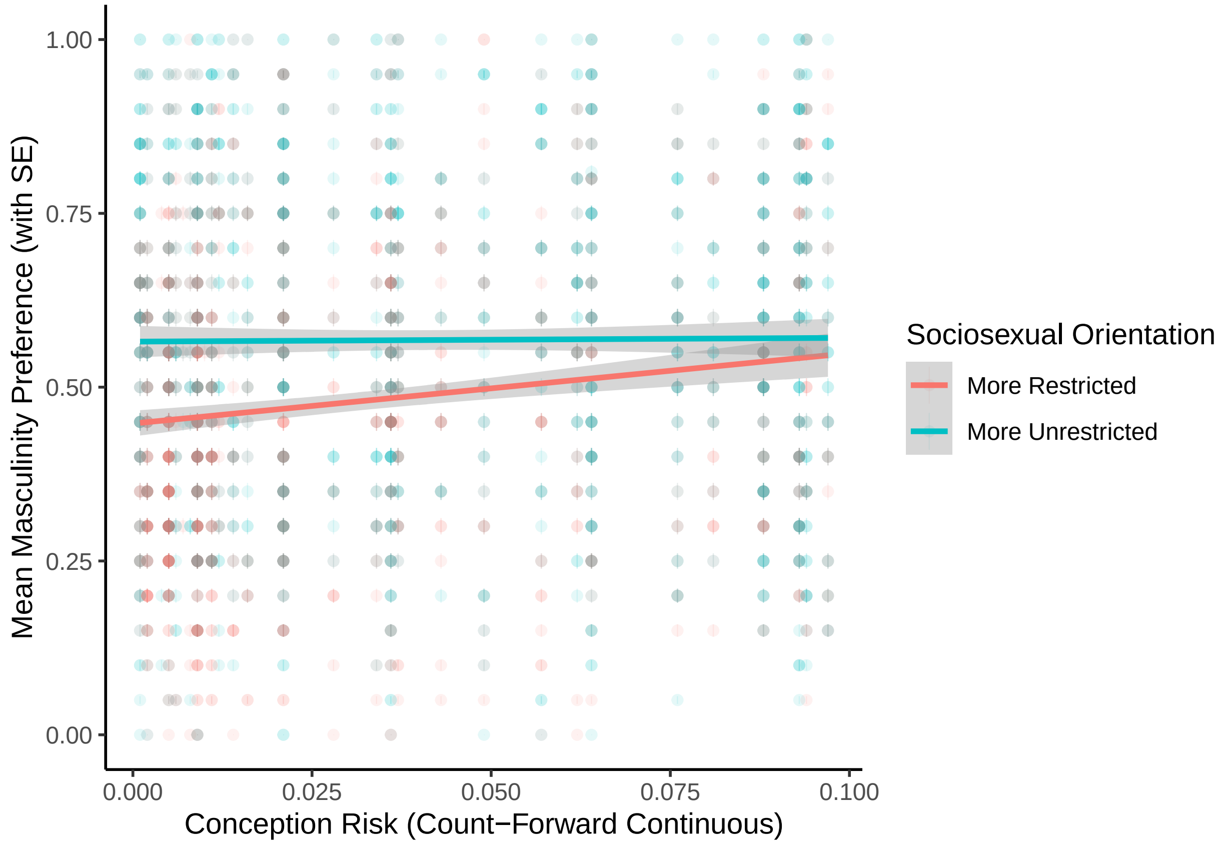


```
ggsave(filename = "fig1.png")
```

Count-Forward Dichotomous

Linear Mixed Effects Model

```
if(file.exists("model.cf_dich.Rdata")){
  load("model.cf_dich.Rdata")
} else {
  model.cf_dich <- glmer(response ~ z.soi*e.cr_cf.dich +
                   (1 | ResponseId) +
                   (1 + z.soi*e.cr_cf.dich || faceId) +
                   (1 + z.soi*e.cr_cf.dich || country) +
                   (1 + z.soi*e.cr_cf.dich || region),data = analysis.data,family = "binomial")
  save(model.cf_dich,file = "model.cf_dich.Rdata")
}

summary(model.cf_dich)
```

```
## Generalized linear mixed model fit by maximum likelihood (Laplace
##   Approximation) [glmerMod]
##  Family: binomial  ( logit )
## Formula: response ~ z.soi * e.cr_cf.dich + (1 | ResponseId) + (1 + z.soi *
##     e.cr_cf.dich || faceId) + (1 + z.soi * e.cr_cf.dich || country) +
##     (1 + z.soi * e.cr_cf.dich || region)
##    Data: analysis.data
##
```

```
##      AIC      BIC   logLik deviance df.resid
##  50896.1  51044.6 -25431.0  50862.1    46064
##
## Scaled residuals:
##     Min      1Q  Median      3Q     Max
## -8.0607 -0.6572  0.2003  0.6469  6.7411
##
## Random effects:
##  Groups     Name                Variance  Std.Dev.
##  ResponseId (Intercept)         1.501e+00 1.225e+00
##  country    z.soi:e.cr_cf.dich 1.476e-10 1.215e-05
##  country.1  e.cr_cf.dich       2.542e-10 1.594e-05
##  country.2  z.soi              3.761e-14 1.939e-07
##  country.3  (Intercept)        7.324e-02 2.706e-01
##  faceId     z.soi:e.cr_cf.dich 4.579e-04 2.140e-02
##  faceId.1   e.cr_cf.dich       6.502e-03 8.063e-02
##  faceId.2   z.soi              3.652e-02 1.911e-01
##  faceId.3   (Intercept)        1.015e+00 1.007e+00
##  region     z.soi:e.cr_cf.dich 1.432e-02 1.197e-01
##  region.1   e.cr_cf.dich       1.255e-10 1.120e-05
##  region.2   z.soi              1.140e-09 3.376e-05
##  region.3   (Intercept)        1.836e-01 4.284e-01
## Number of obs: 46081, groups:
## ResponseId, 2304; country, 25; faceId, 21; region, 7
##
## Fixed effects:
##                    Estimate Std. Error z value Pr(>|z|)
## (Intercept)        0.138708   0.294453   0.471  0.63759
## z.soi              0.146298   0.055085   2.656  0.00791 **
## e.cr_cf.dich       0.092200   0.066984   1.376  0.16869
## z.soi:e.cr_cf.dich 0.007919   0.106891   0.074  0.94094
## ---
## Signif. codes:  0 '***' 0.001 '**' 0.01 '*' 0.05 '.' 0.1 ' ' 1
##
## Correlation of Fixed Effects:
##             (Intr) z.soi  e.cr_.
## z.soi        0.005
## e.cr_cf.dch  0.033 -0.015
## z.s:.cr_cf. -0.006  0.092  0.159
## optimizer (Nelder_Mead) convergence code: 0 (OK)
## boundary (singular) fit: see ?isSingular
```

Plot Data

```
plot.data <- analysis.data %>%
  mutate(soi_group = factor(ifelse(soi >= median(soi),"More Unrestricted","More Restricted"),levels = c
         cr_cf.dich = factor(cr_cf.dich,levels = c("Low","High"))) %>%
    group_by(ResponseId,cr_cf.dich,soi_group) %>%
    summarise(
                mean.response = mean(response,na.rm = TRUE),
                se.response = sd(response,na.rm = TRUE)/n()
                ) %>%
    filter(!is.na(mean.response))
```

```r
ggplot(plot.data,aes(x = soi_group,y = mean.response,fill = cr_cf.dich)) +
    geom_half_violin(data = filter(plot.data,cr_cf.dich == "Low"),side = "l") +
  geom_half_violin(data = filter(plot.data,cr_cf.dich == "High"),side = "r") +
    stat_summary(data = filter(plot.data,cr_cf.dich == "Low"),position = position_nudge(x = -.15)) +
  stat_summary(data = filter(plot.data,cr_cf.dich == "High"),position = position_nudge(x = .15)) +
    theme_classic() +
    xlab("Sociosexual Orientation") +
    ylab("Mean Masculinity Preference (with SE)") +
  labs(fill = "Conception Risk (Count-Forward Dichotomous)") +
  theme(legend.position = "bottom")
```

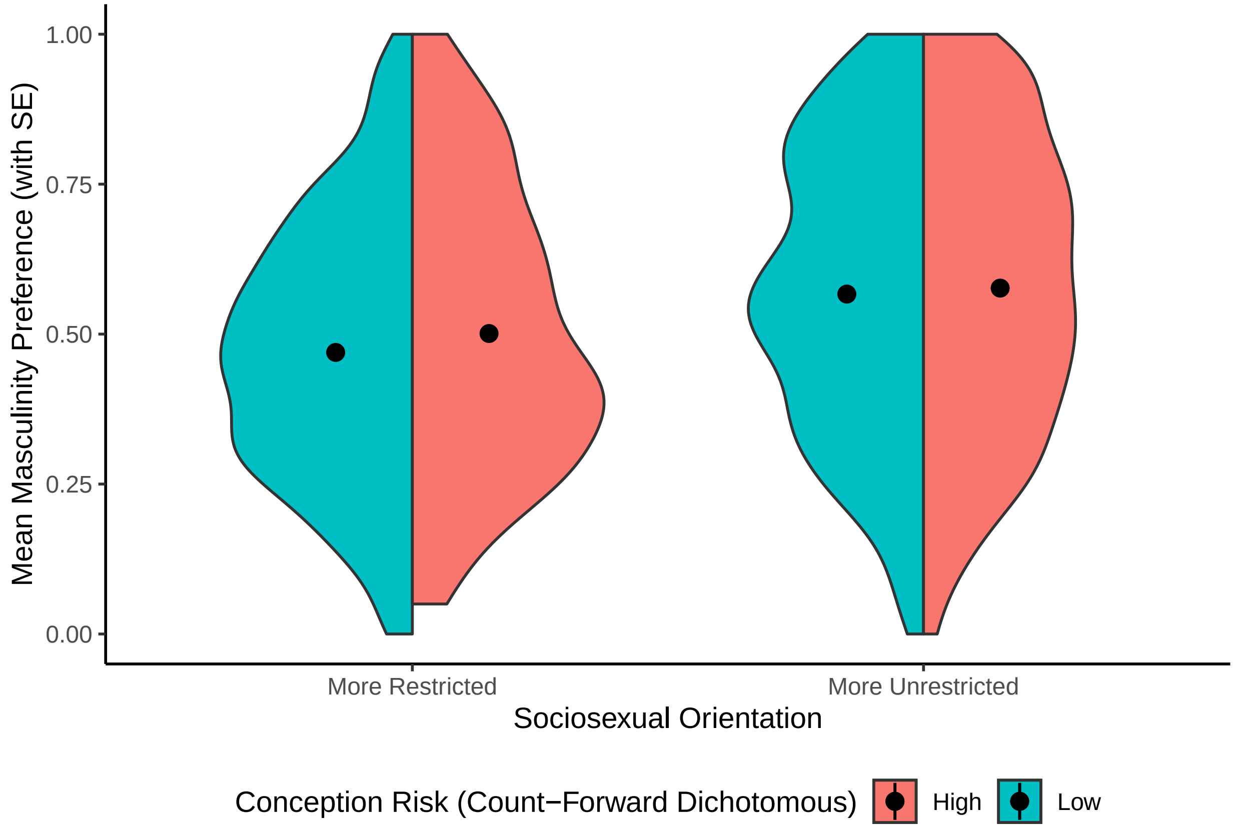


```r
ggsave(filename = "fig2.png")
```

Count-Back - Continuous

Linear Mixed Effects Model

```r
if(file.exists("model.cb_cont.Rdata")){
  load("model.cb_cont.Rdata")
} else {
  model.cb_cont <- glmer(response ~ z.soi*z.cr_cb.cont +
                     (1 | ResponseId) +
                     (1 + z.soi*z.cr_cb.cont || faceId) +
                     (1 + z.soi*z.cr_cb.cont || country) +
                     (1 + z.soi*z.cr_cb.cont || region),data = analysis.data,family = "binomial")
  save(model.cb_cont,file = "model.cb_cont.Rdata")
}
```

```r
summary(model.cb_cont)
```

```
## Generalized linear mixed model fit by maximum likelihood (Laplace
##   Approximation) [glmerMod]
##  Family: binomial  ( logit )
## Formula: response ~ z.soi * z.cr_cb.cont + (1 | ResponseId) + (1 + z.soi *
##     z.cr_cb.cont || faceId) + (1 + z.soi * z.cr_cb.cont || country) +
##     (1 + z.soi * z.cr_cb.cont || region)
##    Data: analysis.data
##
##      AIC      BIC   logLik deviance df.resid
##  49206.6  49354.6 -24586.3  49172.6    44544
##
## Scaled residuals:
##     Min      1Q  Median      3Q     Max
## -8.0060 -0.6565  0.1967  0.6449  6.2491
##
## Random effects:
##  Groups     Name               Variance  Std.Dev.
##  ResponseId (Intercept)        1.501e+00 1.225e+00
##  country    z.soi:z.cr_cb.cont 1.658e-08 1.288e-04
##  country.1  z.cr_cb.cont       9.817e-03 9.908e-02
##  country.2  z.soi              2.029e-10 1.424e-05
##  country.3  (Intercept)        7.935e-02 2.817e-01
##  faceId     z.soi:z.cr_cb.cont 8.228e-03 9.071e-02
##  faceId.1   z.cr_cb.cont       8.521e-03 9.231e-02
##  faceId.2   z.soi              3.163e-02 1.779e-01
##  faceId.3   (Intercept)        1.032e+00 1.016e+00
##  region     z.soi:z.cr_cb.cont 2.018e-10 1.421e-05
##  region.1   z.cr_cb.cont       1.565e-10 1.251e-05
##  region.2   z.soi              4.334e-04 2.082e-02
##  region.3   (Intercept)        1.940e-01 4.404e-01
## Number of obs: 44561, groups:
## ResponseId, 2228; country, 25; faceId, 21; region, 7
##
## Fixed effects:
##                    Estimate Std. Error z value Pr(>|z|)
## (Intercept)         0.14155    0.30158   0.469    0.639
## z.soi               0.14256    0.08735   1.632    0.103
## z.cr_cb.cont        0.01131    0.04722   0.240    0.811
## z.soi:z.cr_cb.cont -0.04396    0.03930  -1.118    0.263
##
## Correlation of Fixed Effects:
##             (Intr) z.soi  z.cr_.
## z.soi        0.047
## z.cr_cb.cnt -0.011 -0.057
## z.s:z.cr_c. -0.004  0.095 -0.021
## optimizer (Nelder_Mead) convergence code: 0 (OK)
## boundary (singular) fit: see ?isSingular
```

Plot Data

```r
plot.data <- analysis.data %>%
  mutate(soi_group = factor(ifelse(soi >= median(soi),"More Unrestricted","More Restricted"),levels = c
    group_by(ResponseId,cr_cb.cont,soi_group) %>%
    summarise(
                mean.response = mean(response,na.rm = TRUE),
                se.response = sd(response,na.rm = TRUE)/n()
                ) %>%
    filter(!is.na(mean.response))

ggplot(plot.data,aes(x = cr_cb.cont,y = mean.response,group = soi_group,colour = soi_group)) +
    geom_pointrange(aes(ymin = mean.response - se.response,ymax = mean.response + se.response),size = .
    geom_smooth(data = plot.data,method = "lm") +
    theme_classic() +
    xlab("Conception Risk (Count-Back Continuous)") +
    ylab("Mean Masculinity Preference (with SE)") +
  labs(colour = "Sociosexual Orientation")
```

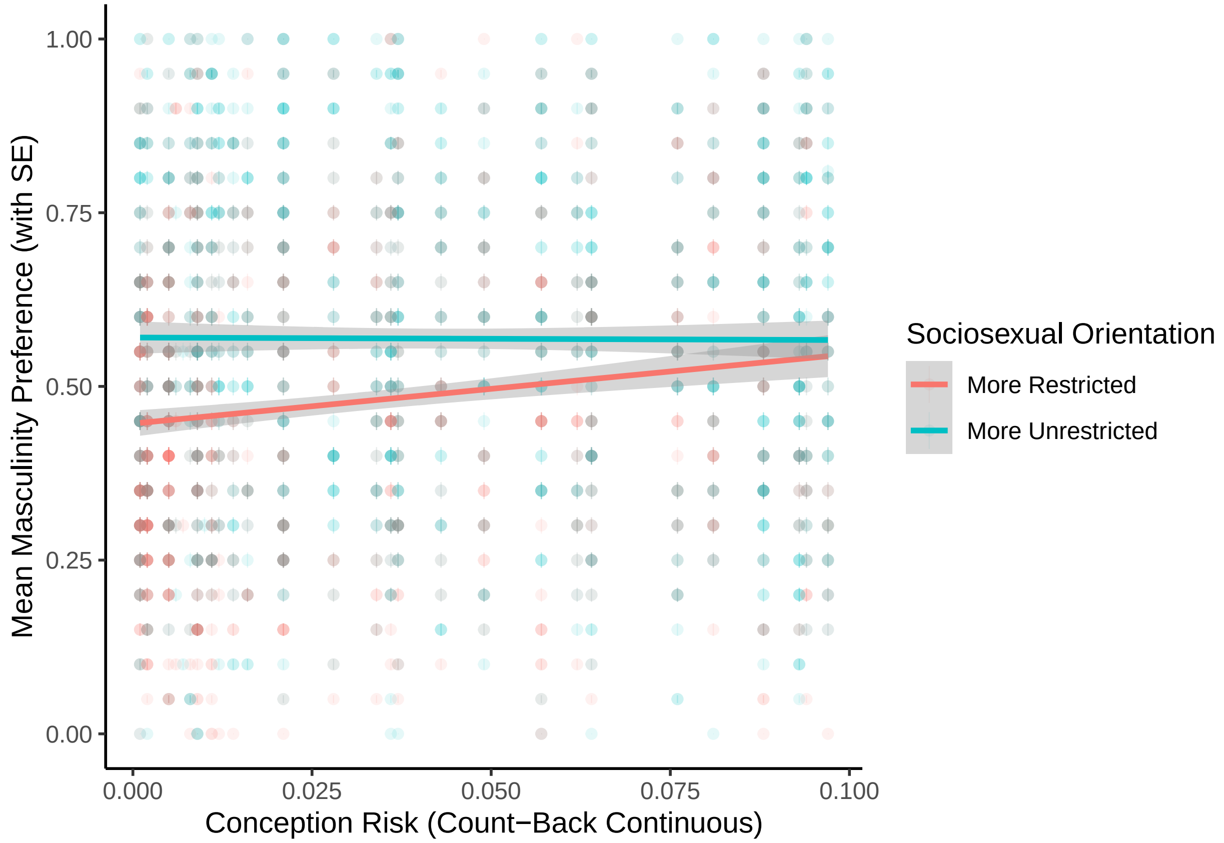


```r
ggsave(filename = "fig3.png")
```

Count-Back Dichotomous

Linear Mixed Effects Model

```r
if(file.exists("model.cb_dich.Rdata")){
  load("model.cb_dich.Rdata")
} else {
```

```r
  model.cb_dich <- glmer(response ~ z.soi*e.cr_cb.dich +
                    (1 | ResponseId) +
                    (1 + z.soi*e.cr_cb.dich || faceId) +
                    (1 + z.soi*e.cr_cb.dich || country) +
                    (1 + z.soi*e.cr_cb.dich || region),data = analysis.data,family = "binomial")
  save(model.cb_dich,file = "model.cb_dich.Rdata")
}

summary(model.cb_dich)
```

```
## Generalized linear mixed model fit by maximum likelihood (Laplace
##   Approximation) [glmerMod]
##  Family: binomial  ( logit )
## Formula: response ~ z.soi * e.cr_cb.dich + (1 | ResponseId) + (1 + z.soi *
##     e.cr_cb.dich || faceId) + (1 + z.soi * e.cr_cb.dich || country) +
##     (1 + z.soi * e.cr_cb.dich || region)
##    Data: analysis.data
##
##      AIC      BIC   logLik deviance df.resid
##  49827.2  49975.4 -24896.6  49793.2    45104
##
## Scaled residuals:
##     Min      1Q  Median      3Q     Max
## -7.9516 -0.6564  0.1993  0.6455  6.5785
##
## Random effects:
##  Groups     Name               Variance  Std.Dev.
##  ResponseId (Intercept)        1.507e+00 1.227e+00
##  country    z.soi:e.cr_cb.dich 5.370e-07 7.328e-04
##  country.1  e.cr_cb.dich       2.779e-08 1.667e-04
##  country.2  z.soi              0.000e+00 0.000e+00
##  country.3  (Intercept)        7.684e-02 2.772e-01
##  faceId     z.soi:e.cr_cb.dich 2.749e-03 5.243e-02
##  faceId.1   e.cr_cb.dich       1.604e-02 1.267e-01
##  faceId.2   z.soi              3.416e-02 1.848e-01
##  faceId.3   (Intercept)        1.029e+00 1.015e+00
##  region     z.soi:e.cr_cb.dich 1.184e-02 1.088e-01
##  region.1   e.cr_cb.dich       1.888e-08 1.374e-04
##  region.2   z.soi              1.331e-09 3.649e-05
##  region.3   (Intercept)        1.949e-01 4.415e-01
## Number of obs: 45121, groups:
## ResponseId, 2256; country, 25; faceId, 21; region, 7
##
## Fixed effects:
##                    Estimate Std. Error z value Pr(>|z|)
## (Intercept)         0.12788    0.30050   0.426  0.67043
## z.soi               0.15388    0.05435   2.831  0.00463 **
## e.cr_cb.dich        0.06451    0.07372   0.875  0.38155
## z.soi:e.cr_cb.dich  0.10830    0.11580   0.935  0.34970
## ---
## Signif. codes:  0 '***' 0.001 '**' 0.01 '*' 0.05 '.' 0.1 ' ' 1
##
## Correlation of Fixed Effects:
##             (Intr) z.soi  e.cr_.
```

```
## z.soi        0.005
## e.cr_cb.dch  0.024 -0.021
## z.s:.cr_cb. -0.019  0.103  0.205
## optimizer (Nelder_Mead) convergence code: 0 (OK)
## boundary (singular) fit: see ?isSingular
```

Plot Data

```
plot.data <- analysis.data %>%
  mutate(soi_group = factor(ifelse(soi >= median(soi),"More Unrestricted","More Restricted"),levels = c
         cr_cb.dich = factor(cr_cb.dich,levels = c("Low","High"))) %>%
    group_by(ResponseId,cr_cb.dich,soi_group) %>%
    summarise(
                mean.response = mean(response,na.rm = TRUE),
                se.response = sd(response,na.rm = TRUE)/n()
                ) %>%
    filter(!is.na(mean.response)) %>%
  filter(!is.na(cr_cb.dich))

ggplot(plot.data,aes(x = soi_group,y = mean.response,fill = cr_cb.dich)) +
    geom_half_violin(data = filter(plot.data,cr_cb.dich == "Low"),side = "l") +
  geom_half_violin(data = filter(plot.data,cr_cb.dich == "High"),side = "r") +
    stat_summary(data = filter(plot.data,cr_cb.dich == "Low"),position = position_nudge(x = -.15)) +
  stat_summary(data = filter(plot.data,cr_cb.dich == "High"),position = position_nudge(x = .15)) +
    theme_classic() +
    xlab("Sociosexual Orientation") +
    ylab("Mean Masculinity Preference (with SE)") +
  labs(fill = "Conception Risk (Count-Back Dichotomous)") +
  theme(legend.position = "bottom")
```

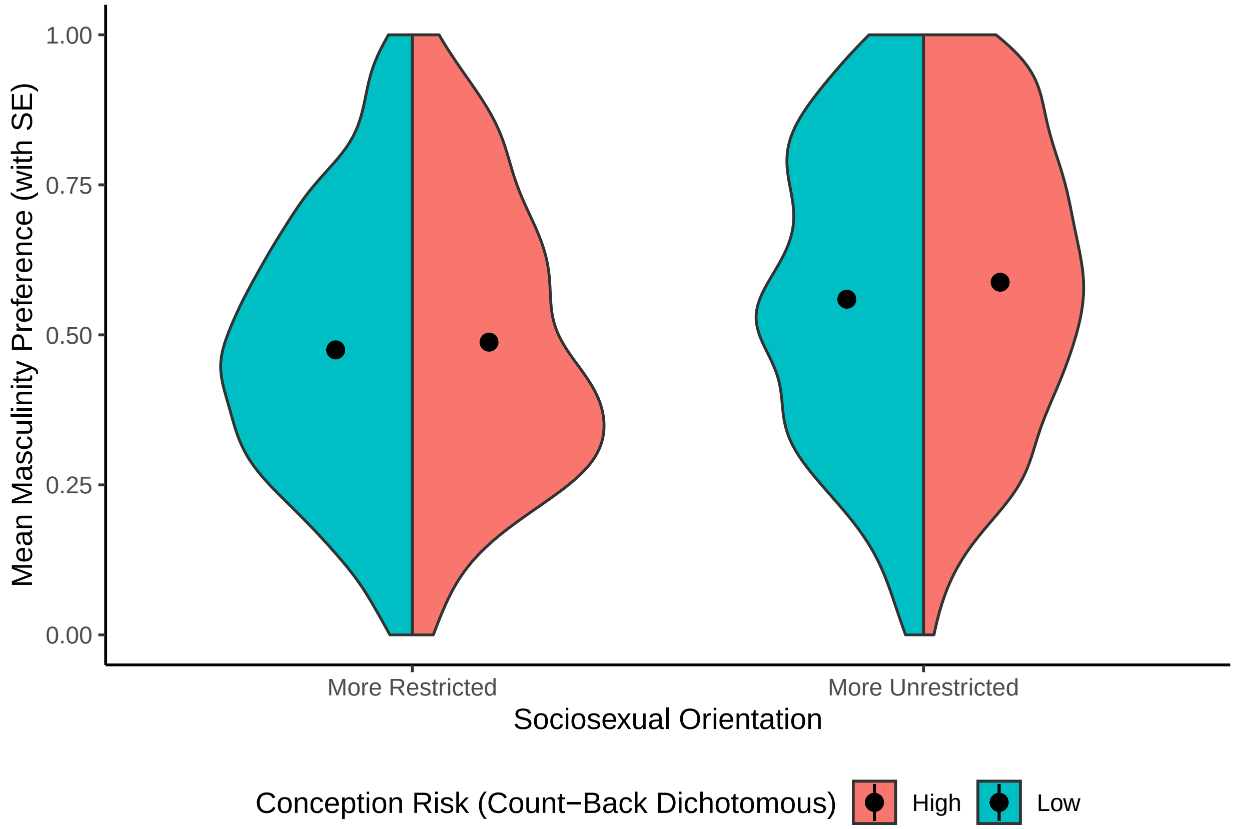


```
ggsave(filename = "fig4.png")
```
