# Supplementary material for: No evidence that sociosexual orientation moderates effects of conception probability on women’s preferences for male facial masculinity
Source: Sci Rep. 2023 Jun 23;13:10245. doi: 10.1038/s41598-023-37404-6 (PMC10290078; doi:10.1038/s41598-023-37404-6)
Supplement: Supplementary file 4 — Supplementary Information 4. [file 41598_2023_37404_MOESM4_ESM.pdf]

Conception Probability and Sociosexual Orientation on Facial Masculinity Preferences - Sample 2

Contents

Load Packages and Custom Functions **1**

Load Data **2**

Participant Demographics **3**

Prepare Data **3**
SOI . . . 3
Conception Risk . . . 4
Facial Masculinity Preference . . . 5
Join Datasets . . . 5

Analyses - Does SOI interact with conception risk to predict facial masculinity preference. **5**
Count-forward - Continuous . . . 5
Count-Forward Dichotomous . . . 7
Count-Back - Continuous . . . 9
Count-Back Dichotomous . . . 11

Load Packages and Custom Functions

```
library(tidyverse)
library(lubridate)
library(lme4)
library(lmerTest)
library(gghalves)

z <- function(x,remove.outliers = FALSE,winsorise = FALSE){
    out <- (x - mean(x,na.rm = TRUE))/sd(x,na.rm = TRUE)
    if (remove.outliers == TRUE){
      out <- ifelse(out >3,NA,ifelse(out < -3,NA,out))
    }
    if (winsorise == TRUE){
      out <- ifelse(out > 3,3,ifelse(out < -3,-3,out))
    }
    return(out)
}

checkN <- function(data,label){
  print(paste(label,": ",NROW(data),sep = ""))
  return(data)
}
```

```
recode.mp <- function(x,reverse = FALSE){
    if(reverse == FALSE){
        out <- recode(x,"The one on the left is much more attractive" = 1,
                    "The one on the left is more attractive" = 2,
                    "The one on the left is somewhat more attractive" = 3,
                    "The one on the left is slightly more attractive" = 4,
                    "The one on the right is slightly more attractive" = 5,
                    "The one on the right is somewhat more attractive" = 6,
                    "The one on the right is more attractive" = 7,
                    "The one on the right is much more attractive" = 8)
    } else {
        out <- recode(x,"The one on the left is much more attractive" = 8,
                    "The one on the left is more attractive" = 7,
                    "The one on the left is somewhat more attractive" = 6,
                    "The one on the left is slightly more attractive" = 5,
                    "The one on the right is slightly more attractive" = 4,
                    "The one on the right is somewhat more attractive" = 3,
                    "The one on the right is more attractive" = 2,
                    "The one on the right is much more attractive" = 1)
    }
    out <- as.numeric(out)
    return(out)
}
```

Load Data

```
data <- read.csv("finnish.data.csv") %>%  checkN("Full Sample") %>%
  filter(gender == "female") %>% checkN("Female") %>%
    filter(SEXORIENT_selfperceived_f == "Heterosexual") %>% checkN("Exclusively Heterosexual") %>%
  filter(ever_pregnant != "I am pregnant right now") %>% checkN("Not Pregnant") %>%
  filter(NEW_nursing != "Yes") %>% checkN("Not Lactating") %>%
  filter(menstrual_cycle_fluctuation != "My menstrual cycle is highly irregular, I never know when the
  filter(contracept == "No") %>% checkN("Not Using Hormonal Contraception") %>%
  filter(!is.na(last_period_day)) %>% checkN("Missing Menstrual Last") %>%
  mutate(menfilter = 1,
           menstrual_cycle_length = ifelse(menstrual_cycle_length == "I do not have periods",NA,menstru
           menstrual_cycle_length = ifelse(menstrual_cycle_length == "less than 22",21,menstrual_cycle_
           menstrual_cycle_length = ifelse(menstrual_cycle_length == "more than 38",39,menstrual_cycle_
           menstrual_cycle_length = as.numeric(menstrual_cycle_length),
           menfilter = ifelse(menstrual_cycle_length > 28 | menstrual_cycle_length < 21,0,menfilter))
      filter(menfilter == 1) %>% checkN("Menstrual Length Out of Bounds") %>%
        mutate(soi1 = z(SOI_NEW_partnerstotal,winsorise = TRUE),
              soi2 = z(SOI_NEW_partn5y,winsorise = TRUE),
               soi3 = z(SOI_NEW_unipartn,winsorise = TRUE),
               soi4 = z(recode(SOI_NEW_fantsex,
                           "Never" = 1,
                           "Once every 2-3 months" = 2,
                           "Once a month" = 3,
                           "Once every two weeks" = 4,
                           "Once a week" = 5,
                           "A few times a week" = 6,
                           "Almost every day" = 7,
```

```
                              "At least once a day" = 8),
                  winsorise = TRUE),
                  soi5 = z(SOI_NEW_sexnolov,winsorise = TRUE) * -1,
                  soi6 = z(SOI_NEW_casual,winsorise = TRUE) * -1,
                  soi7 = z((10 - SOI_NEW_attached),winsorise = TRUE) * -1
        ) %>%
        mutate(soi_total = (soi1 + soi2 + soi3 + soi4 + soi5 + soi6 + soi7)/7) %>%
  filter(!is.na(soi_total)) %>% checkN("Missing SOI")
```

```
## [1] "Full Sample: 2166"
## [1] "Female: 2166"
## [1] "Exclusively Heterosexual: 1918"
## [1] "Not Pregnant: 1796"
## [1] "Not Lactating: 1647"
## [1] "Regular Cycle: 1493"
## [1] "Not Using Hormonal Contraception: 860"
## [1] "Missing Menstrual Last: 836"
## [1] "Menstrual Length Out of Bounds: 519"
## [1] "Missing SOI: 483"
```

Participant Demographics

```
#Number of men and women in full sample
table(data$gender)
```

```
##
## female
##    483
```

```
#Mean and SD for age in full sample
summarise(data, mean.age = mean(age_NEW,na.rm = TRUE),
                sd.age = sd(age_NEW,na.rm = TRUE))
```

```
##   mean.age   sd.age
## 1 34.31056 5.306267
```

Prepare Data

SOI

```
soi.data <- data %>%
    select(number,family_id,SOI_NEW_partners:SOI_NEW_attached) %>%
        mutate(soi1 = z(SOI_NEW_partnerstotal,winsorise = TRUE),
               soi2 = z(SOI_NEW_partn5y,winsorise = TRUE),
                soi3 = z(SOI_NEW_unipartn,winsorise = TRUE),
                soi4 = z(recode(SOI_NEW_fantsex,
                            "Never" = 1,
                            "Once every 2-3 months" = 2,
                            "Once a month" = 3,
                            "Once every two weeks" = 4,
                            "Once a week" = 5,
                            "A few times a week" = 6,
                            "Almost every day" = 7,
```

```
                         "At least once a day" = 8),
              winsorise = TRUE),
              soi5 = z(SOI_NEW_sexnolov,winsorise = TRUE) * -1,
              soi6 = z(SOI_NEW_casual,winsorise = TRUE) * -1,
              soi7 = z((10 - SOI_NEW_attached),winsorise = TRUE) * -1
        ) %>%
        mutate(soi_total = (soi1 + soi2 + soi3 + soi4 + soi5 + soi6 + soi7)/7) %>%
        mutate(z.soi = c(scale(soi_total))) %>%
        select(number,family_id,soi_total,z.soi)
```

Conception Risk

```
conception.risk <- read.csv("conception.risk.csv")

cycle.data <- data %>%
    select(number,timestamp,menarche:ever_pregnant) %>%
    mutate(menfilter = 1,
           menfilter = ifelse(contracept == "No",menfilter,0),
           menstrual_cycle_length = ifelse(menstrual_cycle_length == "I do not have periods",NA,menstru
           menstrual_cycle_length = ifelse(menstrual_cycle_length == "less than 22",21,menstrual_cycle_
           menstrual_cycle_length = ifelse(menstrual_cycle_length == "more than 38",39,menstrual_cycle_
           menstrual_cycle_length = as.numeric(menstrual_cycle_length),
           menfilter = ifelse(menstrual_cycle_length > 28 | menstrual_cycle_length < 21,0,menfilter),
           #menfilter = ifelse(!(menstrual_cycle_fluctuation == "My cycle is almost always the same le
           #                    menstrual_cycle_fluctuation == "My cycle is usually around the same le
           menfilter = ifelse(contrastop != "No" & !is.na(contrastop),0,menfilter)
           ) %>%
    filter(menfilter == 1) %>%

    mutate( last_period_year = ifelse(last_period_year == 0,NA,last_period_year),
           last_period_year = ifelse(last_period_year == 13,2013,last_period_year),
           last_period_year = ifelse(last_period_year == 203,2013,last_period_year),
           last_period_year = ifelse(last_period_year == 1012,2012,last_period_year),
           last_period_year = ifelse(last_period_year == 1913,2013,last_period_year),
           last_period_month = ifelse(last_period_month == 0,NA,last_period_month),
           last_period_month = ifelse(last_period_month == 40,NA,last_period_month),
           last_period_day = ifelse(last_period_day == 0,NA,last_period_day),
           last_period = ymd(paste(last_period_year,last_period_month,last_period_day,sep = "-")),
           timestamp = mdy(substr(timestamp,1,10)),
           count.forward = interval(last_period,timestamp) %/% days(1)) %>%
    left_join(conception.risk,by = c("count.forward" = "day")) %>%
    rename(cr.cf_cont = conception.risk) %>%
    mutate( count.back = 28 - interval(timestamp,(last_period + ddays(menstrual_cycle_length))) %/% days
    left_join(conception.risk,by = c("count.back" = "day")) %>%
    rename(cr.cb_cont = conception.risk) %>%
    mutate( cr.cf_dich = ifelse(count.forward >=6 & count.forward <=14,"High","Low"),
           cr.cf_dich = ifelse(is.na(cr.cf_cont),NA,cr.cf_dich),
           cr.cb_dich = ifelse(count.back >= 6 & count.back <=14,"High","Low"),
           cr.cb_dich = ifelse(is.na(cr.cb_cont),NA,cr.cb_dich)) %>%
    mutate(z.cr_cf.cont = c(scale(cr.cf_cont)),
         e.cr_cf.dich = recode(cr.cf_dich,"High" = .5,"Low" = -.5),
         z.cr_cb.cont = c(scale(cr.cb_cont)),
         e.cr_cb.dich = recode(cr.cb_dich,"High" = .5,"Low" = -.5)) %>%
```

```
    select(number,cr.cf_cont,cr.cf_dich,cr.cb_cont,cr.cb_dich,z.cr_cf.cont:e.cr_cb.dich)
```

Facial Masculinity Preference

```
rating.data <- data %>%
    select(number,PICTURES_1:PICTURES_21) %>%
    mutate(PICTURES_1 = recode.mp(PICTURES_1),
            PICTURES_2 = recode.mp(PICTURES_2,reverse = TRUE),
            PICTURES_3 = recode.mp(PICTURES_3,reverse = TRUE),
            PICTURES_4 = recode.mp(PICTURES_4,reverse = TRUE),
            PICTURES_5 = recode.mp(PICTURES_5),
            PICTURES_6 = recode.mp(PICTURES_6,reverse = TRUE),
            PICTURES_7 = recode.mp(PICTURES_7),
            PICTURES_8 = recode.mp(PICTURES_8),
            PICTURES_9 = recode.mp(PICTURES_9,reverse = TRUE),
            PICTURES_10 = recode.mp(PICTURES_10,reverse = TRUE),
            PICTURES_11 = recode.mp(PICTURES_11),
            PICTURES_12 = recode.mp(PICTURES_12,reverse = TRUE),
            PICTURES_13 = recode.mp(PICTURES_13,reverse = TRUE),
            PICTURES_14 = recode.mp(PICTURES_14,reverse = TRUE),
            PICTURES_15 = recode.mp(PICTURES_15),
            PICTURES_16 = recode.mp(PICTURES_16),
            PICTURES_17 = recode.mp(PICTURES_17,reverse = TRUE),
            PICTURES_18 = recode.mp(PICTURES_18,reverse = TRUE),
            PICTURES_19 = recode.mp(PICTURES_19),
            PICTURES_20 = recode.mp(PICTURES_20,reverse = TRUE),
            PICTURES_21 = recode.mp(PICTURES_21,reverse = TRUE),
    ) %>%
    gather(key = "faceId",value = "response",PICTURES_1:PICTURES_21)
```

Join Datasets

```
participant.data <- left_join(soi.data,cycle.data,by = "number") %>%
      filter(!is.na(cr.cf_cont) | !is.na(cr.cb_cont))

analysis.data <- left_join(participant.data,rating.data,by = "number")

write.csv(analysis.data,"analysis.data2.csv",row.names = FALSE)
```

Analyses - Does SOI interact with conception risk to predict facial masculinity preference.

Count-forward - Continuous

Linear Mixed Effects Model

```
if(file.exists("model.cf_cont2.Rdata")){
      load("model.cf_cont2.Rdata")
    } else {
      model.cf_cont <- lmer(response ~ z.soi*z.cr_cf.cont +
                             (1 | number) +
```

```
                              (1 + z.soi*z.cr_cf.cont || family_id) +
                              (1 + z.soi*z.cr_cf.cont || faceId),data = analysis.data)
      save(model.cf_cont,file = "model.cf_cont2.Rdata")
    }

    summary(model.cf_cont)
```

```
## Linear mixed model fit by REML. t-tests use Satterthwaite's method [
## lmerModLmerTest]
## Formula: response ~ z.soi * z.cr_cf.cont + (1 | number) + (1 + z.soi *
##     z.cr_cf.cont || family_id) + (1 + z.soi * z.cr_cf.cont ||      faceId)
##    Data: analysis.data
##
## REML criterion at convergence: 32583.8
##
## Scaled residuals:
##     Min      1Q  Median      3Q     Max
## -4.4707 -0.5152  0.0710  0.6190  4.5526
##
## Random effects:
##  Groups      Name               Variance  Std.Dev.
##  number      (Intercept)        0.3979126 0.63080
##  family_id   (Intercept)        0.0934914 0.30576
##  family_id.1 z.soi              0.0032041 0.05660
##  family_id.2 z.cr_cf.cont       0.0000000 0.00000
##  family_id.3 z.soi:z.cr_cf.cont 0.0784441 0.28008
##  faceId      (Intercept)        0.3753684 0.61267
##  faceId.1    z.soi              0.0008711 0.02951
##  faceId.2    z.cr_cf.cont       0.0038168 0.06178
##  faceId.3    z.soi:z.cr_cf.cont 0.0000000 0.00000
##  Residual                       1.8776355 1.37027
## Number of obs: 9114, groups:  number, 434; family_id, 410; faceId, 21
##
## Fixed effects:
##                     Estimate Std. Error        df t value Pr(>|t|)
## (Intercept)          5.24987    0.13917  22.94527  37.722   <2e-16 ***
## z.soi                0.04511    0.04219  43.13878   1.069   0.2909
## z.cr_cf.cont        -0.08010    0.04180 195.90094  -1.916   0.0568 .
## z.soi:z.cr_cf.cont   0.02584    0.04644 121.78030   0.557   0.5789
## ---
## Signif. codes:  0 '***' 0.001 '**' 0.01 '*' 0.05 '.' 0.1 ' ' 1
##
## Correlation of Fixed Effects:
##             (Intr) z.soi  z.cr_.
## z.soi        0.011
## z.cr_cf.cnt  0.005 -0.046
## z.s:z.cr_c. -0.014 -0.006  0.061
## optimizer (nloptwrap) convergence code: 0 (OK)
## boundary (singular) fit: see help('isSingular')
```

Plot Data

```
plot.data <- analysis.data %>%
      mutate(soi_group = factor(ifelse(soi_total >= median(soi_total,na.rm = TRUE),"More Unrestricted",
      group_by(number,cr.cf_cont,soi_group) %>%
      summarise(
        mean.response = mean(response,na.rm = TRUE),
        se.response = sd(response,na.rm = TRUE)/n()
      ) %>%
      filter(!is.na(mean.response),!is.na(soi_group))

    ggplot(plot.data,aes(x = cr.cf_cont,y = mean.response,group = soi_group,colour = soi_group)) +
      geom_pointrange(aes(ymin = mean.response - se.response,ymax = mean.response + se.response),size =
      geom_smooth(data = plot.data,method = "lm") +
      theme_classic() +
      xlab("Conception Risk (Count-Forward Continuous)") +
      ylab("Mean Masculinity Preference (with SE)") +
      labs(colour = "Sociosexual Orientation")
```

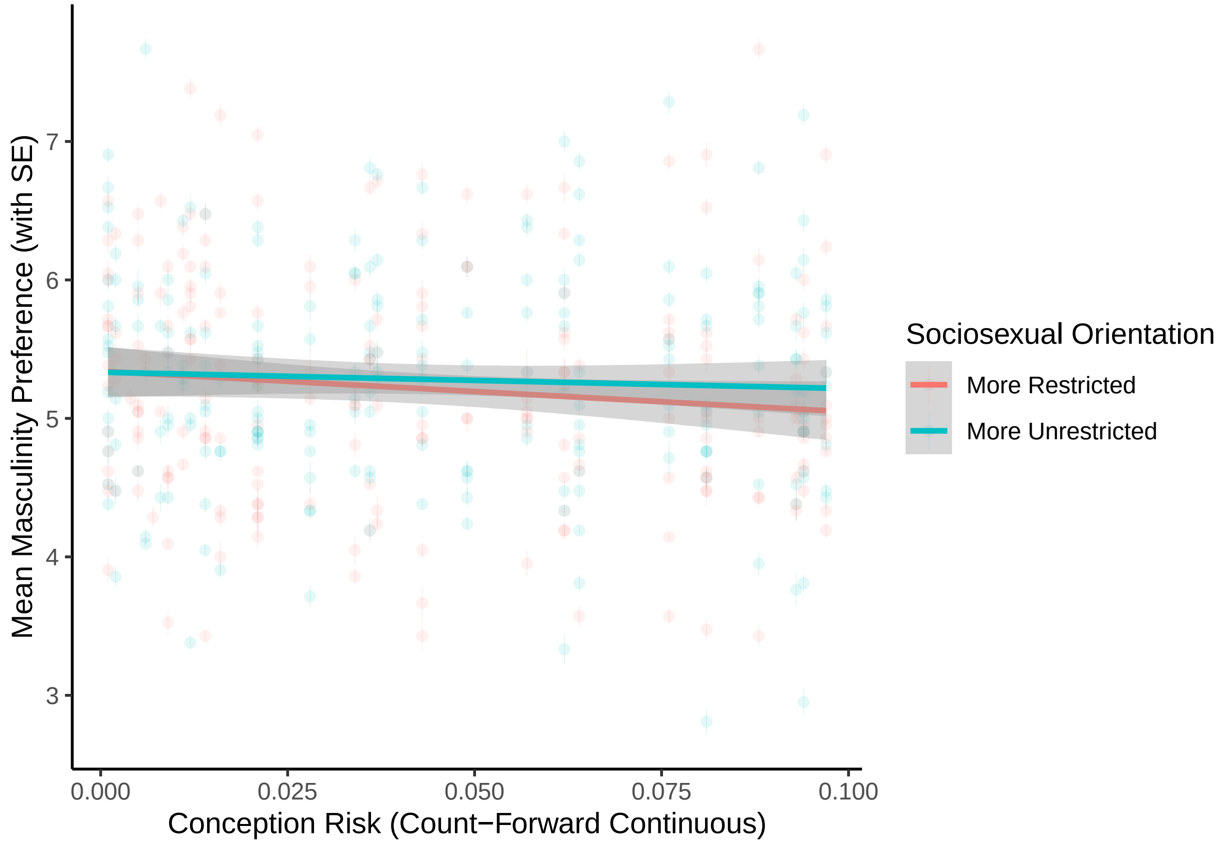


```
    ggsave(filename = "fig1.png")
```

Count-Forward Dichotomous

Linear Mixed Effects Model

```
    if(file.exists("model.cf_dich2.Rdata")){
      load("model.cf_dich2.Rdata")
    } else {
      model.cf_dich <- lmer(response ~ z.soi*e.cr_cf.dich +
```

```r
                              (1 | number) +
                              (1 + z.soi*e.cr_cf.dich || family_id) +
                              (1 + z.soi*e.cr_cf.dich || faceId),data = analysis.data)
      save(model.cf_dich,file = "model.cf_dich2.Rdata")
    }

    summary(model.cf_dich)
```

```
## Linear mixed model fit by REML. t-tests use Satterthwaite's method [
## lmerModLmerTest]
## Formula: response ~ z.soi * e.cr_cf.dich + (1 | number) + (1 + z.soi *
##     e.cr_cf.dich || family_id) + (1 + z.soi * e.cr_cf.dich ||      faceId)
##    Data: analysis.data
##
## REML criterion at convergence: 32586.9
##
## Scaled residuals:
##     Min      1Q  Median      3Q     Max
## -4.4339 -0.5134  0.0649  0.6159  4.5851
##
## Random effects:
##  Groups      Name                Variance  Std.Dev.
##  number      (Intercept)         3.524e-01 5.936e-01
##  family_id   (Intercept)         5.184e-02 2.277e-01
##  family_id.1 z.soi               9.777e-02 3.127e-01
##  family_id.2 e.cr_cf.dich        2.950e-01 5.431e-01
##  family_id.3 z.soi:e.cr_cf.dich 1.460e-09 3.821e-05
##  faceId      (Intercept)         3.740e-01 6.116e-01
##  faceId.1    z.soi               7.469e-04 2.733e-02
##  faceId.2    e.cr_cf.dich        8.980e-03 9.476e-02
##  faceId.3    z.soi:e.cr_cf.dich 0.000e+00 0.000e+00
##  Residual                        1.879e+00 1.371e+00
## Number of obs: 9114, groups:  number, 434; family_id, 410; faceId, 21
##
## Fixed effects:
##                     Estimate Std. Error        df t value Pr(>|t|)
## (Intercept)          5.23819    0.13940  23.24576  37.576   <2e-16 ***
## z.soi                0.05519    0.04641 131.26623   1.189   0.2365
## e.cr_cf.dich        -0.13975    0.08329 246.91251  -1.678   0.0946 .
## z.soi:e.cr_cf.dich   0.02822    0.09170 144.39861   0.308   0.7587
## ---
## Signif. codes:  0 '***' 0.001 '**' 0.01 '*' 0.05 '.' 0.1 ' ' 1
##
## Correlation of Fixed Effects:
##             (Intr) z.soi  e.cr_.
## z.soi        0.014
## e.cr_cf.dch  0.075 -0.021
## z.s:.cr_cf. -0.006  0.251  0.052
## optimizer (nloptwrap) convergence code: 0 (OK)
## boundary (singular) fit: see help('isSingular')
```

Plot Data

```
plot.data <- analysis.data %>%
      mutate(soi_group = factor(ifelse(soi_total >= median(soi_total,na.rm = TRUE),"More Unrestricted",
             cr_cf.dich = factor(cr.cf_dich,levels = c("Low","High"))) %>%
      group_by(number,cr_cf.dich,soi_group) %>%
      summarise(
        mean.response = mean(response,na.rm = TRUE),
        se.response = sd(response,na.rm = TRUE)/n()
      ) %>%
      filter(!is.na(mean.response),!is.na(soi_group))

    ggplot(plot.data,aes(x = soi_group,y = mean.response,fill = cr_cf.dich)) +
      geom_half_violin(data = filter(plot.data,cr_cf.dich == "Low"),side = "l") +
      geom_half_violin(data = filter(plot.data,cr_cf.dich == "High"),side = "r") +
      stat_summary(data = filter(plot.data,cr_cf.dich == "Low"),position = position_nudge(x = -.15)) +
      stat_summary(data = filter(plot.data,cr_cf.dich == "High"),position = position_nudge(x = .15)) +
      theme_classic() +
      xlab("Sociosexual Orientation") +
      ylab("Mean Masculinity Preference (with SE)") +
      labs(fill = "Conception Risk (Count-Forward Dichotomous)") +
      theme(legend.position = "bottom")
```

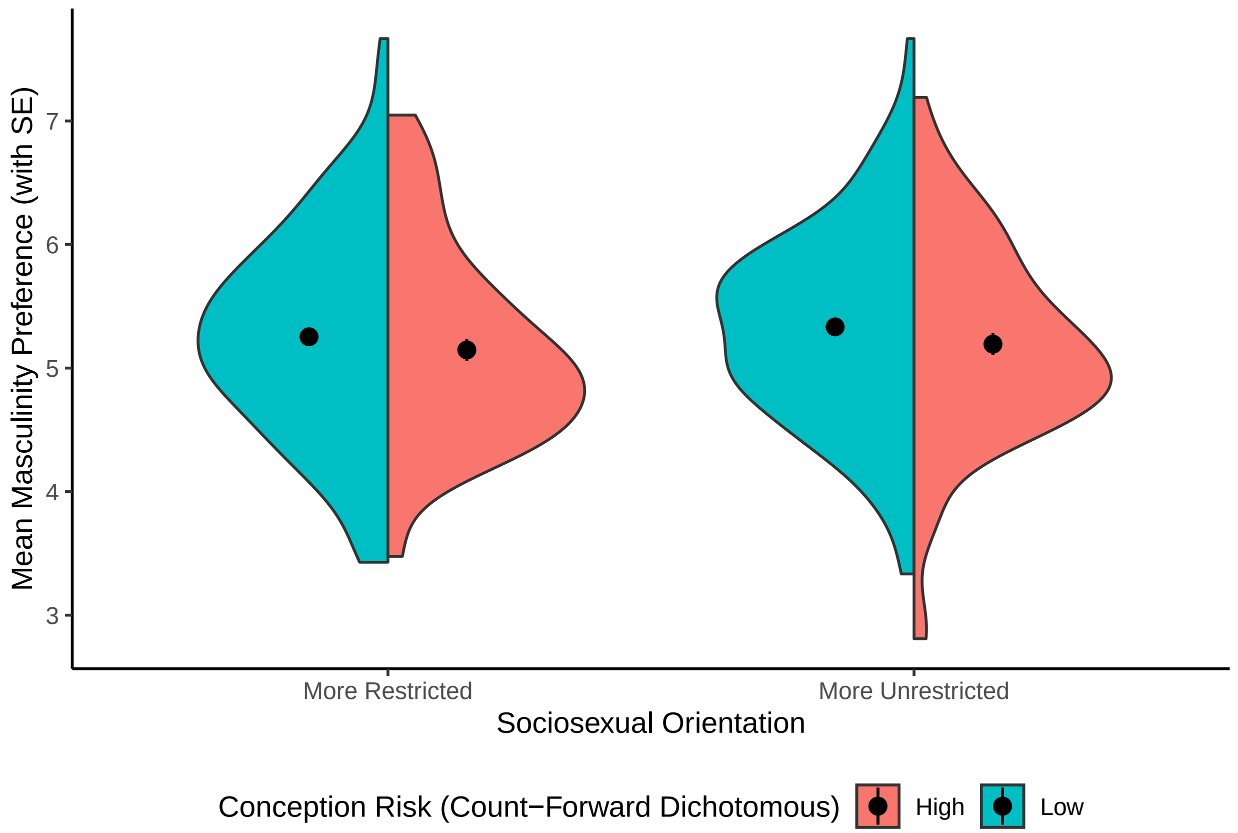


```
    ggsave(filename = "fig2.png")
```

Count-Back - Continuous

Linear Mixed Effects Model

```r
if(file.exists("model.cb_cont2.Rdata")){
  load("model.cb_cont2.Rdata")
} else {
  model.cb_cont <- lmer(response ~ z.soi*z.cr_cb.cont +
                          (1 | number) +
                          (1 + z.soi*z.cr_cb.cont || family_id) +
                          (1 + z.soi*z.cr_cb.cont || faceId),data = analysis.data)
  save(model.cb_cont,file = "model.cb_cont2.Rdata")
}

summary(model.cb_cont)
```

```
## Linear mixed model fit by REML. t-tests use Satterthwaite's method [
## lmerModLmerTest]
## Formula: response ~ z.soi * z.cr_cb.cont + (1 | number) + (1 + z.soi *
##     z.cr_cb.cont || family_id) + (1 + z.soi * z.cr_cb.cont ||      faceId)
##    Data: analysis.data
##
## REML criterion at convergence: 33196.7
##
## Scaled residuals:
##     Min      1Q  Median      3Q     Max
## -4.4482 -0.5143  0.0698  0.6214  4.5789
##
## Random effects:
##  Groups      Name               Variance Std.Dev.
##  number      (Intercept)        0.365828 0.60484
##  family_id   (Intercept)        0.122592 0.35013
##  family_id.1 z.soi              0.041829 0.20452
##  family_id.2 z.cr_cb.cont       0.000000 0.00000
##  family_id.3 z.soi:z.cr_cb.cont 0.040928 0.20231
##  faceId      (Intercept)        0.379167 0.61577
##  faceId.1    z.soi              0.000459 0.02142
##  faceId.2    z.cr_cb.cont       0.002694 0.05190
##  faceId.3    z.soi:z.cr_cb.cont 0.000000 0.00000
##  Residual                       1.881197 1.37157
## Number of obs: 9282, groups:  number, 442; family_id, 417; faceId, 21
##
## Fixed effects:
##                     Estimate Std. Error         df t value Pr(>|t|)
## (Intercept)         5.257888   0.139799  22.883654  37.610   <2e-16 ***
## z.soi               0.060756   0.043505  98.797238   1.397   0.1657
## z.cr_cb.cont       -0.075234   0.040483 225.602775  -1.858   0.0644 .
## z.soi:z.cr_cb.cont -0.002507   0.044536 105.301217  -0.056   0.9552
## ---
## Signif. codes:  0 '***' 0.001 '**' 0.01 '*' 0.05 '.' 0.1 ' ' 1
##
## Correlation of Fixed Effects:
##             (Intr) z.soi  z.cr_.
## z.soi        0.018
## z.cr_cb.cnt  0.004 -0.029
## z.s:z.cr_c. -0.008  0.020  0.030
```

```
## optimizer (nloptwrap) convergence code: 0 (OK)
## boundary (singular) fit: see help('isSingular')
```

Plot Data

```
plot.data <- analysis.data %>%
      mutate(soi_group = factor(ifelse(soi_total >= median(soi_total,na.rm = TRUE),"More Unrestricted",
      group_by(number,cr.cb_cont,soi_group) %>%
      summarise(
        mean.response = mean(response,na.rm = TRUE),
        se.response = sd(response,na.rm = TRUE)/n()
      ) %>%
      filter(!is.na(mean.response),!is.na(soi_group))

    ggplot(plot.data,aes(x = cr.cb_cont,y = mean.response,group = soi_group,colour = soi_group)) +
      geom_pointrange(aes(ymin = mean.response - se.response,ymax = mean.response + se.response),size =
      geom_smooth(data = plot.data,method = "lm") +
      theme_classic() +
      xlab("Conception Risk (Count-Back Continuous)") +
      ylab("Mean Masculinity Preference (with SE)") +
      labs(colour = "Sociosexual Orientation")
```

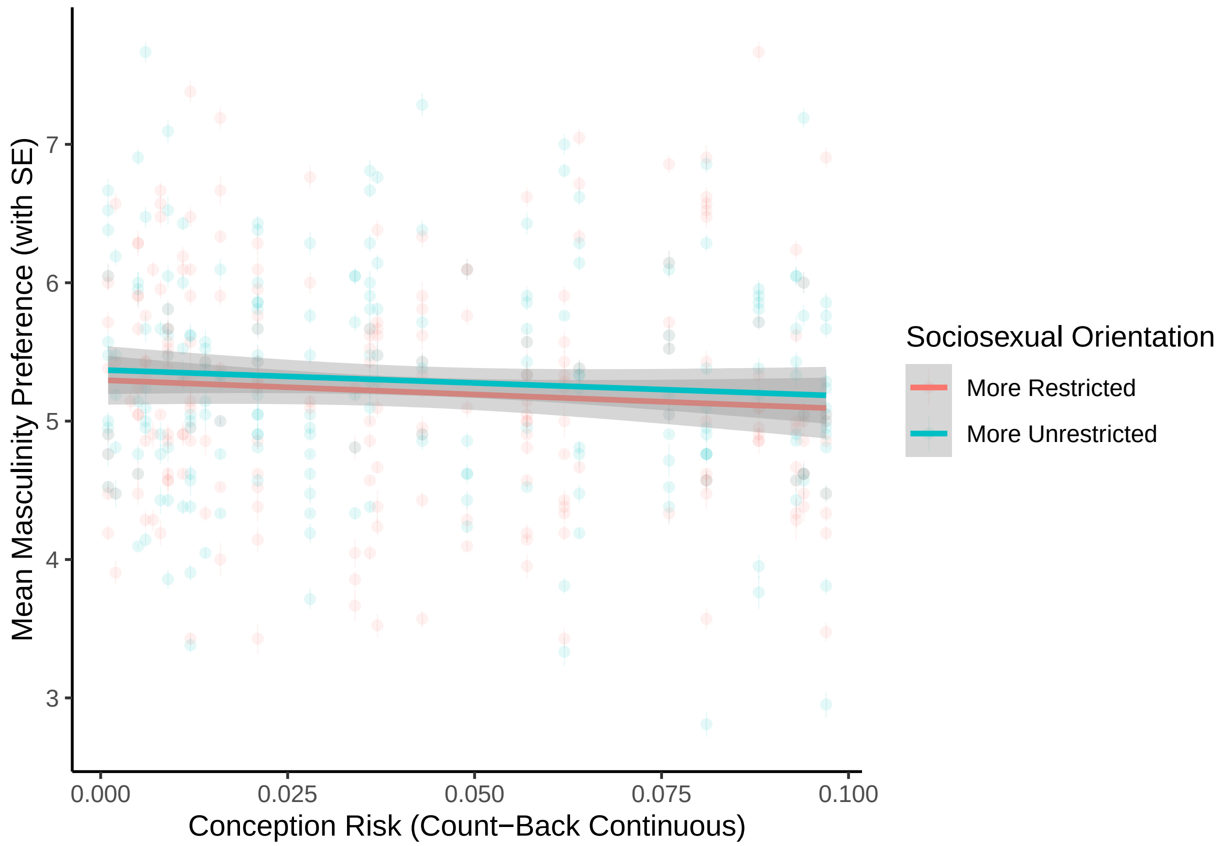


```
    ggsave(filename = "fig3.png")
```

Count-Back Dichotomous

Linear Mixed Effects Model

```
if(file.exists("model.cb_dich2.Rdata")){
  load("model.cb_dich2.Rdata")
} else {
  model.cb_dich <- lmer(response ~ z.soi*e.cr_cb.dich +
                         (1 | number) +
                         (1 + z.soi*e.cr_cb.dich || family_id) +
                         (1 + z.soi*e.cr_cb.dich || faceId),data = analysis.data)
  save(model.cb_dich,file = "model.cb_dich2.Rdata")
}

summary(model.cb_dich)
```

```
## Linear mixed model fit by REML. t-tests use Satterthwaite's method [
## lmerModLmerTest]
## Formula: response ~ z.soi * e.cr_cb.dich + (1 | number) + (1 + z.soi *
##     e.cr_cb.dich || family_id) + (1 + z.soi * e.cr_cb.dich ||      faceId)
##    Data: analysis.data
##
## REML criterion at convergence: 33199.3
##
## Scaled residuals:
##     Min      1Q  Median      3Q     Max
## -4.4222 -0.5138  0.0689  0.6208  4.6164
##
## Random effects:
##  Groups      Name               Variance  Std.Dev.
##  number      (Intercept)        3.289e-01 5.735e-01
##  family_id   (Intercept)        6.501e-02 2.550e-01
##  family_id.1 z.soi              9.235e-02 3.039e-01
##  family_id.2 e.cr_cb.dich       3.664e-01 6.053e-01
##  family_id.3 z.soi:e.cr_cb.dich 6.023e-09 7.761e-05
##  faceId      (Intercept)        3.772e-01 6.142e-01
##  faceId.1    z.soi              3.122e-04 1.767e-02
##  faceId.2    e.cr_cb.dich       4.850e-03 6.964e-02
##  faceId.3    z.soi:e.cr_cb.dich 0.000e+00 0.000e+00
##  Residual                       1.883e+00 1.372e+00
## Number of obs: 9282, groups:  number, 442; family_id, 417; faceId, 21
##
## Fixed effects:
##                     Estimate Std. Error         df t value Pr(>|t|)
## (Intercept)         5.249718   0.140154  23.354453  37.457   <2e-16 ***
## z.soi               0.055728   0.047143 131.317097   1.182    0.239
## e.cr_cb.dich       -0.063745   0.083435 268.388586  -0.764    0.446
## z.soi:e.cr_cb.dich -0.007352   0.093044 156.707848  -0.079    0.937
## ---
## Signif. codes:  0 '***' 0.001 '**' 0.01 '*' 0.05 '.' 0.1 ' ' 1
##
## Correlation of Fixed Effects:
##             (Intr) z.soi e.cr_.
## z.soi       0.016
## e.cr_cb.dch 0.097  0.010
## z.s:.cr_cb. 0.002  0.350 0.054
```

```
## optimizer (nloptwrap) convergence code: 0 (OK)
## boundary (singular) fit: see help('isSingular')
```

Plot Data

```
plot.data <- analysis.data %>%
      mutate(soi_group = factor(ifelse(soi_total >= median(soi_total,na.rm = TRUE),"More Unrestricted",
             cr_cb.dich = factor(cr.cb_dich,levels = c("Low","High"))) %>%
      group_by(number,cr_cb.dich,soi_group) %>%
      summarise(
        mean.response = mean(response,na.rm = TRUE),
        se.response = sd(response,na.rm = TRUE)/n()
      ) %>%
      filter(!is.na(mean.response)) %>%
      filter(!is.na(cr_cb.dich),!is.na(soi_group))

    ggplot(plot.data,aes(x = soi_group,y = mean.response,fill = cr_cb.dich)) +
      geom_half_violin(data = filter(plot.data,cr_cb.dich == "Low"),side = "l") +
      geom_half_violin(data = filter(plot.data,cr_cb.dich == "High"),side = "r") +
      stat_summary(data = filter(plot.data,cr_cb.dich == "Low"),position = position_nudge(x = -.15)) +
      stat_summary(data = filter(plot.data,cr_cb.dich == "High"),position = position_nudge(x = .15)) +
      theme_classic() +
      xlab("Sociosexual Orientation") +
      ylab("Mean Masculinity Preference (with SE)") +
      labs(fill = "Conception Risk (Count-Back Dichotomous)") +
      theme(legend.position = "bottom")
```

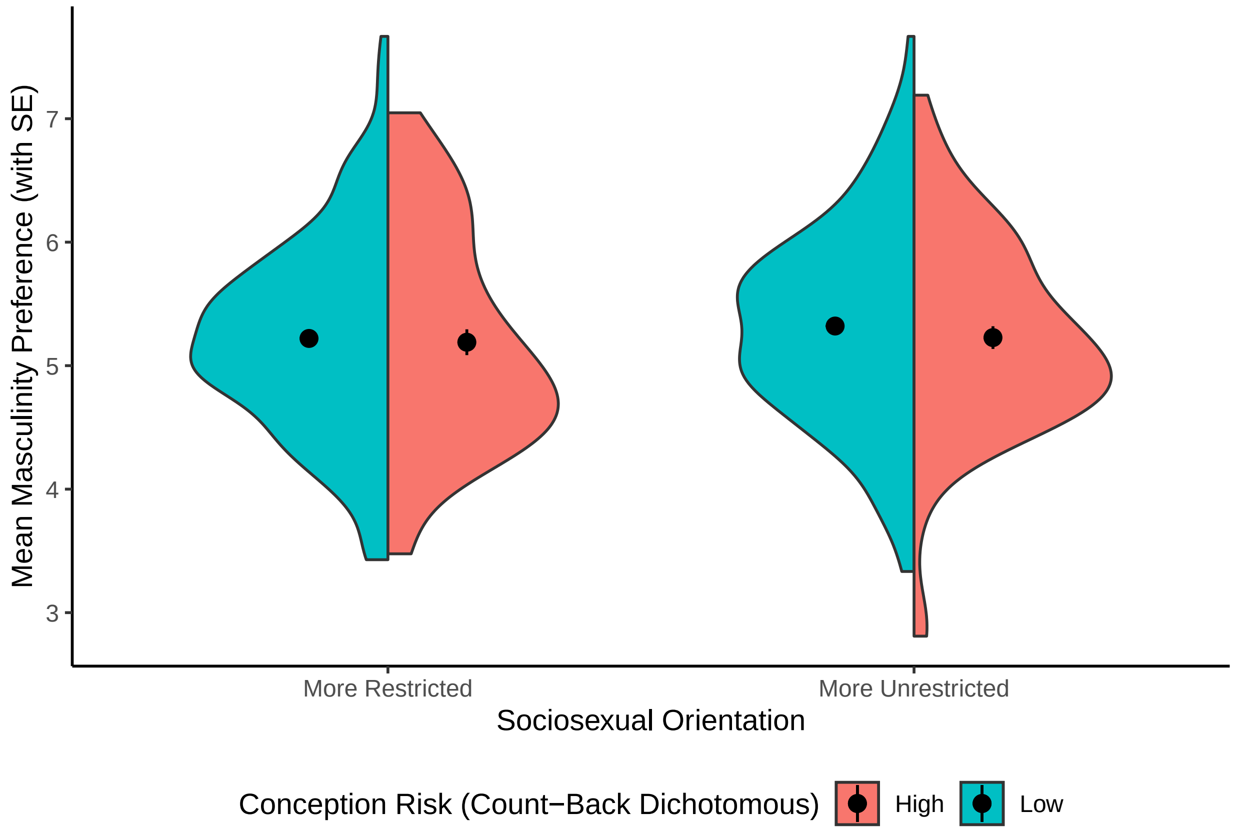

```
ggsave(filename = "fig4.png")
```
