# Supplementary material for: No evidence that sociosexual orientation moderates effects of conception probability on women’s preferences for male facial masculinity
Source: Sci Rep. 2023 Jun 23;13:10245. doi: 10.1038/s41598-023-37404-6 (PMC10290078; doi:10.1038/s41598-023-37404-6)
Supplement: Supplementary file 5 — Supplementary Information 5. [file 41598_2023_37404_MOESM5_ESM.pdf]

Conception Probability and Sociosexual Orientation on Facial Masculinity Preferences - Sample 3

Contents

Load Packages and Custom Functions 1

Load and Prepare Data 1
Participant Demographics . . . 3
Join Datasets . . . 3

Analyses - Does SOI interact with conception risk to predict facial masculinity preference. 3
Count-forward - Continuous . . . 3
Count-Forward Dichotomous . . . 5
Count-Back - Continuous . . . 7
Count-Back Dichotomous . . . 9

Load Packages and Custom Functions

```
library(tidyverse)
library(lme4)
library(lmerTest)
library(gghalves)

z <- function(x,remove.outliers = FALSE,winsorise = FALSE){
    out <- (x - mean(x,na.rm = TRUE))/sd(x,na.rm = TRUE)
    if (remove.outliers == TRUE){
      out <- ifelse(out >3,NA,ifelse(out < -3,NA,out))
    }
    if (winsorise == TRUE){
      out <- ifelse(out > 3,3,ifelse(out < -3,-3,out))
    }
    return(out)
}

checkN <- function(data,label){
  print(paste(label,": ",NROW(data),sep = ""))
  return(data)
}
```

Load and Prepare Data

```
data <- read_csv("data_sample3.csv")

afc.data <- data %>%
```

```
  select(random.id,Blazej:afc_ivan2) %>%
  gather(key = "image.id",value = "response",Blazej:afc_ivan2) %>%
  filter(!is.na(response)) %>%
  rename(ResponseId = random.id) %>%
  rename(faceId = image.id)

individual.data <-
  select(data,ResponseId = random.id,end.date = EndDate,sex,age,sexual.orientation,Hormone_contracept:Re
         soi2 = one_night_stand,
         soi3 = sex_wo_interest,
         soi4 = opinion_sex_wo_love_1,
         soi5 = comfort_w_casual_sex_1,
         soi6 = no_sex_until_LTR_1,
         soi7 = fantasies_wo_LTR,
         soi8 = arousal_wo_LTR,
         soi9 = fantasy_of_stranger) %>% checkN("Full Sample") %>%
  filter(sex == 2) %>% checkN("Female") %>%
  mutate(soi = soi1 + soi2 + soi3 + soi4 + soi5 + ((soi6*-1) + 10) + soi7 + soi8 + soi9) %>%
  filter(!is.na(soi)) %>% checkN("Missing SOI") %>%
  filter(grepl("1",sexual.orientation) | grepl("3",sexual.orientation) | grepl("4",sexual.orientation))
  filter(Pregnant == 2) %>% checkN("Not Pregnant") %>%
  filter(breastfeeding == 2) %>% checkN("Not Lactating") %>%
  filter(Regular_cycle == 1) %>% checkN("Regular Cycle") %>%
  filter(Hormone_contracept == 2) %>% checkN("Not Using Hormonal Contraception") %>%
  rename(menstrual.last = Day_Since_Last_Bleed) %>%
  mutate(menstrual.last = as.numeric(menstrual.last)) %>%
  #filter(!is.na(menstrual.last)) %>% checkN("Missing Menstrual Last") %>%
  rename(menstrual.length = Length_of_cycle) %>%
  mutate(menstrual.length = menstrual.length + 23) %>%
  filter(menstrual.length <= 38) %>% checkN("Menstrual Length Out of Bounds") %>%
  left_join(read.csv("conception.risk.csv",stringsAsFactors = FALSE),by = c("menstrual.last" = "day")) 
  select(ResponseId,sex,age,menstrual.length,menstrual.last,soi,cr_cf.cont = conception.risk) %>%
  mutate(cr_cf.dich = ifelse(menstrual.last >= 6 & menstrual.last <= 14,"High","Low")) %>%
  mutate(count.back = 28 - (menstrual.length - menstrual.last),
         count.back = ifelse(count.back < 0,NA,count.back),
         cr_cb.dich = ifelse(count.back >=6 & count.back <= 14,"High","Low"),
         cr_cb.dich = ifelse(is.na(count.back),NA,cr_cb.dich)) %>%
  left_join(read.csv("conception.risk.csv",stringsAsFactors = FALSE),by = c("count.back" = "day")) %>%
  select(ResponseId,sex,age,soi,cr_cf.cont:cr_cb.dich,cr_cb.cont = conception.risk,-count.back) %>%
  mutate(z.soi = c(scale(soi)),
         z.cr_cf.cont = c(scale(cr_cf.cont)),
         e.cr_cf.dich = recode(cr_cf.dich,"High" = .5,"Low" = -.5),
         z.cr_cb.cont = c(scale(cr_cb.cont)),
         e.cr_cb.dich = recode(cr_cb.dich,"High" = .5,"Low" = -.5))
```

```
## [1] "Full Sample: 1507"
## [1] "Female: 1463"
## [1] "Missing SOI: 1043"
## [1] "Exclusively Heterosexual: 1026"
## [1] "Not Pregnant: 934"
## [1] "Not Lactating: 924"
## [1] "Regular Cycle: 584"
## [1] "Not Using Hormonal Contraception: 346"
## [1] "Menstrual Length Out of Bounds: 339"
```

Participant Demographics

```
#Number of men and women in full sample
table(individual.data$sex)
```

```
##
##   2
## 339
```

```
#Mean and SD for age in full sample
summarise(individual.data,
          mean.age = mean(age,na.rm = TRUE),
          sd.age = sd(age,na.rm = TRUE))
```

```
## # A tibble: 1 x 2
##   mean.age sd.age
##      <dbl>  <dbl>
## 1     25.4   7.57
```

Join Datasets

```
analysis.data <-left_join(afc.data,individual.data,by = "ResponseId")

write.csv(analysis.data,"analysis.data_sample3.csv",row.names = FALSE)
```

Analyses - Does SOI interact with conception risk to predict facial masculinity preference.

Count-forward - Continuous

Linear Mixed Effects Model

```
if(file.exists("model.cf_cont.Rdata")){
  load("model.cf_cont.Rdata")
} else {
  model.cf_cont <- glmer(response ~ z.soi*z.cr_cf.cont +
                         (1 | ResponseId) +
                         (1 + z.soi*z.cr_cf.cont || faceId),data = analysis.data,family = "binomial")
  save(model.cf_cont,file = "model.cf_cont.Rdata")
}

summary(model.cf_cont)
```

```
## Generalized linear mixed model fit by maximum likelihood (Laplace
##   Approximation) [glmerMod]
##  Family: binomial  ( logit )
## Formula: response ~ z.soi * z.cr_cf.cont + (1 | ResponseId) + (1 + z.soi *
##     z.cr_cf.cont || faceId)
##    Data: analysis.data
##
##      AIC      BIC   logLik deviance df.resid
##  14745.0  14812.0  -7363.5  14727.0    12680
##
## Scaled residuals:
```

```
##      Min       1Q  Median      3Q     Max
## -4.2199 -0.8525  0.4161  0.6817  6.8649
##
## Random effects:
##  Groups     Name               Variance  Std.Dev.
##  ResponseId (Intercept)        5.146e-01 7.174e-01
##  faceId     (Intercept)        7.455e-01 8.634e-01
##  faceId.1   z.soi              5.011e-09 7.079e-05
##  faceId.2   z.cr_cf.cont       3.766e-03 6.137e-02
##  faceId.3   z.soi:z.cr_cf.cont 4.588e-03 6.773e-02
## Number of obs: 12689, groups:  ResponseId, 305; faceId, 42
##
## Fixed effects:
##                    Estimate Std. Error z value Pr(>|z|)
## (Intercept)         0.67133    0.14111   4.758 1.96e-06 ***
## z.soi               0.08329    0.04477   1.860   0.0628 .
## z.cr_cf.cont        0.02277    0.04695   0.485   0.6277
## z.soi:z.cr_cf.cont  0.01048    0.04744   0.221   0.8251
## ---
## Signif. codes:  0 '***' 0.001 '**' 0.01 '*' 0.05 '.' 0.1 ' ' 1
##
## Correlation of Fixed Effects:
##             (Intr) z.soi  z.cr_.
## z.soi        0.001
## z.cr_cf.cnt -0.002  0.003
## z.s:z.cr_c.  0.001  0.026  0.024
## optimizer (Nelder_Mead) convergence code: 0 (OK)
## boundary (singular) fit: see ?isSingular
```

Plot Data

```
plot.data <- analysis.data %>%
  mutate(soi_group = factor(ifelse(soi >= median(soi,na.rm = TRUE),"More Unrestricted","More Restricted
  group_by(ResponseId,cr_cf.cont,soi_group) %>%
  summarise(
    mean.response = mean(response,na.rm = TRUE),
    se.response = sd(response,na.rm = TRUE)/n()
  ) %>%
  filter(!is.na(mean.response)) %>%
  filter(!is.na(soi_group))

ggplot(plot.data,aes(x = cr_cf.cont,y = mean.response,group = soi_group,colour = soi_group)) +
  geom_pointrange(aes(ymin = mean.response - se.response,ymax = mean.response + se.response),size = .2,
  geom_smooth(data = plot.data,method = "lm") +
  theme_classic() +
  xlab("Conception Risk (Count-Forward Continuous)") +
  ylab("Mean Masculinity Preference (with SE)") +
  labs(colour = "Sociosexual Orientation")
```

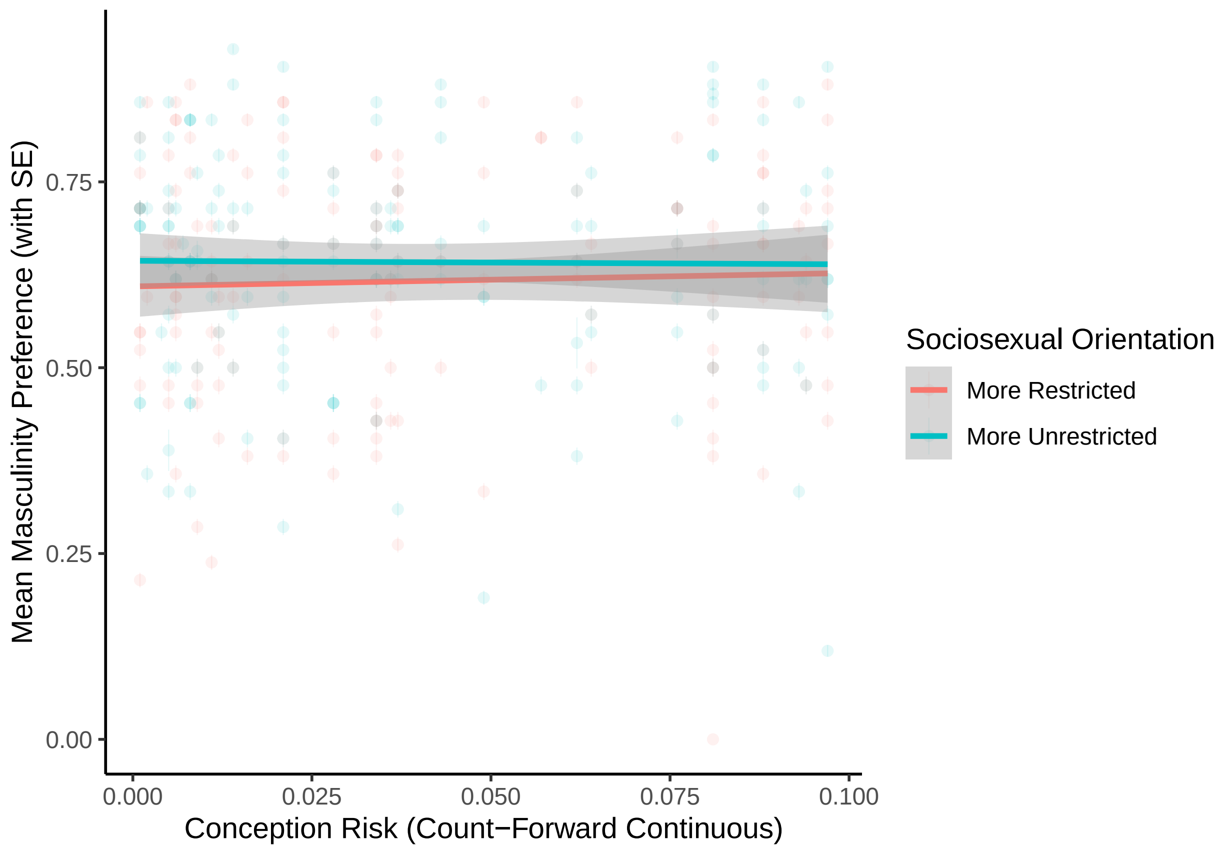


```
ggsave(filename = "fig1.png")
```

Count-Forward Dichotomous

Linear Mixed Effects Model

```
if(file.exists("model.cf_dich.Rdata")){
  load("model.cf_dich.Rdata")
} else {
  model.cf_dich <- glmer(response ~ z.soi*e.cr_cf.dich +
                         (1 | ResponseId) +
                         (1 + z.soi*e.cr_cf.dich || faceId),data = analysis.data,family = "binomial")
  save(model.cf_dich,file = "model.cf_dich.Rdata")
}

summary(model.cf_dich)
```

```
## Generalized linear mixed model fit by maximum likelihood (Laplace
##   Approximation) [glmerMod]
##  Family: binomial  ( logit )
## Formula: response ~ z.soi * e.cr_cf.dich + (1 | ResponseId) + (1 + z.soi *
##     e.cr_cf.dich || faceId)
##    Data: analysis.data
##
##      AIC      BIC   logLik deviance df.resid
##  14744.0  14811.1  -7363.0  14726.0    12680
##
```

```
## Scaled residuals:
##     Min      1Q  Median      3Q     Max
## -4.2641 -0.8505  0.4157  0.6822  6.8061
##
## Random effects:
##  Groups     Name               Variance Std.Dev.
##  ResponseId (Intercept)        0.51491  0.7176
##  faceId     (Intercept)        0.74555  0.8635
##  faceId.1   z.soi              0.00000  0.0000
##  faceId.2   e.cr_cf.dich       0.00000  0.0000
##  faceId.3   z.soi:e.cr_cf.dich 0.02894  0.1701
## Number of obs: 12689, groups:  ResponseId, 305; faceId, 42
##
## Fixed effects:
##                     Estimate Std. Error z value Pr(>|z|)
## (Intercept)         0.667757   0.142177   4.697 2.64e-06 ***
## z.soi               0.083170   0.046793   1.777   0.0755 .
## e.cr_cf.dich       -0.020130   0.098485  -0.204   0.8380
## z.soi:e.cr_cf.dich -0.000963   0.097322  -0.010   0.9921
## ---
## Signif. codes:  0 '***' 0.001 '**' 0.01 '*' 0.05 '.' 0.1 ' ' 1
##
## Correlation of Fixed Effects:
##             (Intr) z.soi  e.cr_.
## z.soi        0.000
## e.cr_cf.dch  0.123 -0.004
## z.s:.cr_cf. -0.002  0.280 -0.001
## optimizer (Nelder_Mead) convergence code: 0 (OK)
## boundary (singular) fit: see ?isSingular
```

Plot Data

```
plot.data <- analysis.data %>%
  mutate(soi_group = factor(ifelse(soi >= median(soi,na.rm = TRUE),"More Unrestricted","More Restricted
         cr_cf.dich = factor(cr_cf.dich,levels = c("Low","High"))) %>%
  group_by(ResponseId,cr_cf.dich,soi_group) %>%
  summarise(
    mean.response = mean(response,na.rm = TRUE),
    se.response = sd(response,na.rm = TRUE)/n()
  ) %>%
  filter(!is.na(mean.response)) %>%
  filter(!is.na(soi_group))

ggplot(plot.data,aes(x = soi_group,y = mean.response,fill = cr_cf.dich)) +
  geom_half_violin(data = filter(plot.data,cr_cf.dich == "Low"),side = "l") +
  geom_half_violin(data = filter(plot.data,cr_cf.dich == "High"),side = "r") +
  stat_summary(data = filter(plot.data,cr_cf.dich == "Low"),position = position_nudge(x = -.15)) +
  stat_summary(data = filter(plot.data,cr_cf.dich == "High"),position = position_nudge(x = .15)) +
  theme_classic() +
  xlab("Sociosexual Orientation") +
  ylab("Mean Masculinity Preference (with SE)") +
  labs(fill = "Conception Risk (Count-Forward Dichotomous)") +
  theme(legend.position = "bottom")
```

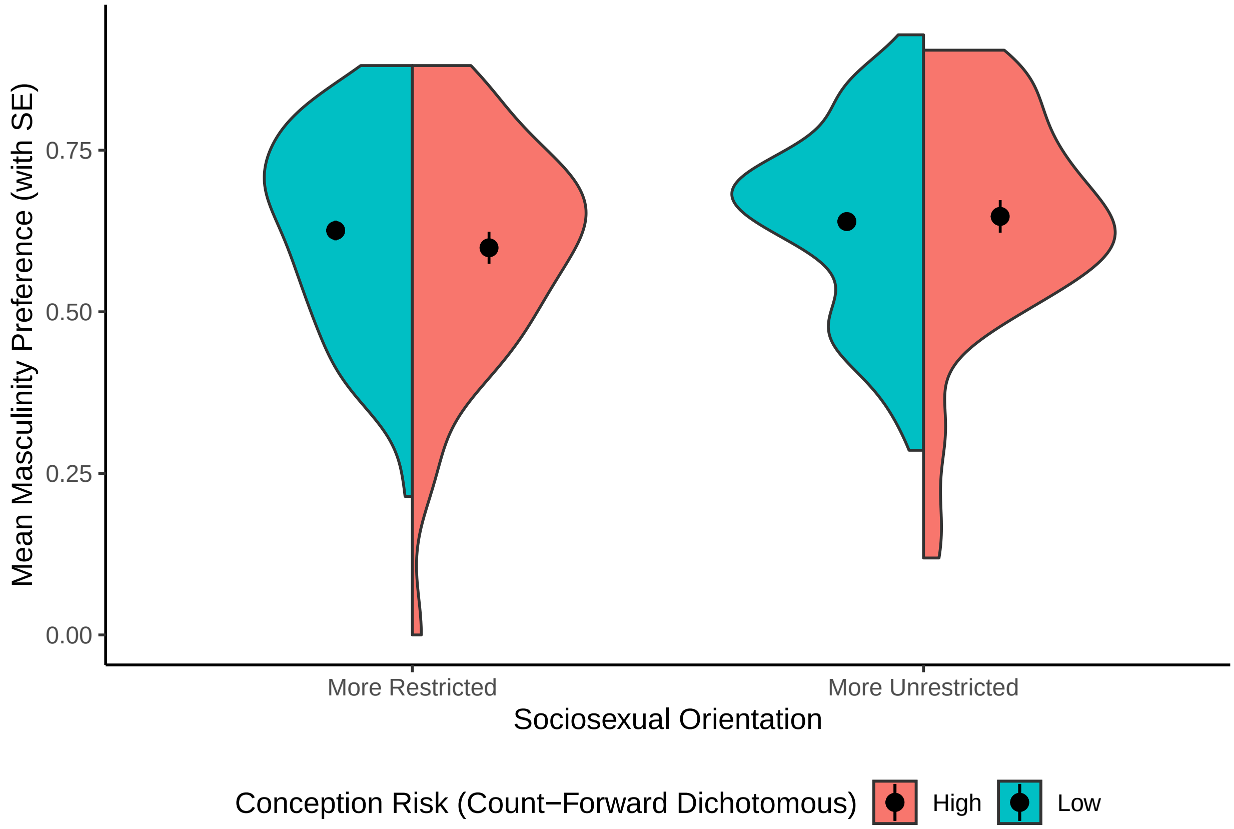


```
ggsave(filename = "fig2.png")
```

Count-Back - Continuous

Linear Mixed Effects Model

```
if(file.exists("model.cb_cont.Rdata")){
  load("model.cb_cont.Rdata")
} else {
  model.cb_cont <- glmer(response ~ z.soi*z.cr_cb.cont +
                         (1 | ResponseId) +
                         (1 + z.soi*z.cr_cb.cont || faceId),data = analysis.data,family = "binomial")
  save(model.cb_cont,file = "model.cb_cont.Rdata")
}

summary(model.cb_cont)
```

```
## Generalized linear mixed model fit by maximum likelihood (Laplace
##   Approximation) [glmerMod]
##  Family: binomial  ( logit )
## Formula: response ~ z.soi * z.cr_cb.cont + (1 | ResponseId) + (1 + z.soi *
##     z.cr_cb.cont || faceId)
##    Data: analysis.data
##
##      AIC      BIC   logLik deviance df.resid
##  14280.7  14347.5  -7131.4  14262.7    12260
##
```

```
## Scaled residuals:
##     Min      1Q  Median      3Q     Max
## -4.1633 -0.8557  0.4175  0.6841  6.7498
##
## Random effects:
##  Groups     Name               Variance  Std.Dev.
##  ResponseId (Intercept)        5.001e-01 7.072e-01
##  faceId     (Intercept)        7.361e-01 8.580e-01
##  faceId.1   z.soi              3.979e-09 6.308e-05
##  faceId.2   z.cr_cb.cont       4.091e-03 6.396e-02
##  faceId.3   z.soi:z.cr_cb.cont 7.439e-03 8.625e-02
## Number of obs: 12269, groups:  ResponseId, 295; faceId, 42
##
## Fixed effects:
##                     Estimate Std. Error z value Pr(>|z|)
## (Intercept)         0.672881   0.140395   4.793 1.64e-06 ***
## z.soi               0.072905   0.045169   1.614    0.107
## z.cr_cb.cont        0.074965   0.047430   1.581    0.114
## z.soi:z.cr_cb.cont -0.002109   0.048265  -0.044    0.965
## ---
## Signif. codes:  0 '***' 0.001 '**' 0.01 '*' 0.05 '.' 0.1 ' ' 1
##
## Correlation of Fixed Effects:
##             (Intr) z.soi  z.cr_.
## z.soi        0.000
## z.cr_cb.cnt  0.003 -0.028
## z.s:z.cr_c. -0.010  0.093  0.026
## optimizer (Nelder_Mead) convergence code: 0 (OK)
## boundary (singular) fit: see ?isSingular
```

Plot Data

```
plot.data <- analysis.data %>%
  mutate(soi_group = factor(ifelse(soi >= median(soi,na.rm = TRUE),"More Unrestricted","More Restricted
  group_by(ResponseId,cr_cb.cont,soi_group) %>%
  summarise(
    mean.response = mean(response,na.rm = TRUE),
    se.response = sd(response,na.rm = TRUE)/n()
  ) %>%
  filter(!is.na(mean.response)) %>%
  filter(!is.na(soi_group))

ggplot(plot.data,aes(x = cr_cb.cont,y = mean.response,group = soi_group,colour = soi_group)) +
  geom_pointrange(aes(ymin = mean.response - se.response,ymax = mean.response + se.response),size = .2,a
  geom_smooth(data = plot.data,method = "lm") +
  theme_classic() +
  xlab("Conception Risk (Count-Back Continuous)") +
  ylab("Mean Masculinity Preference (with SE)") +
  labs(colour = "Sociosexual Orientation")
```

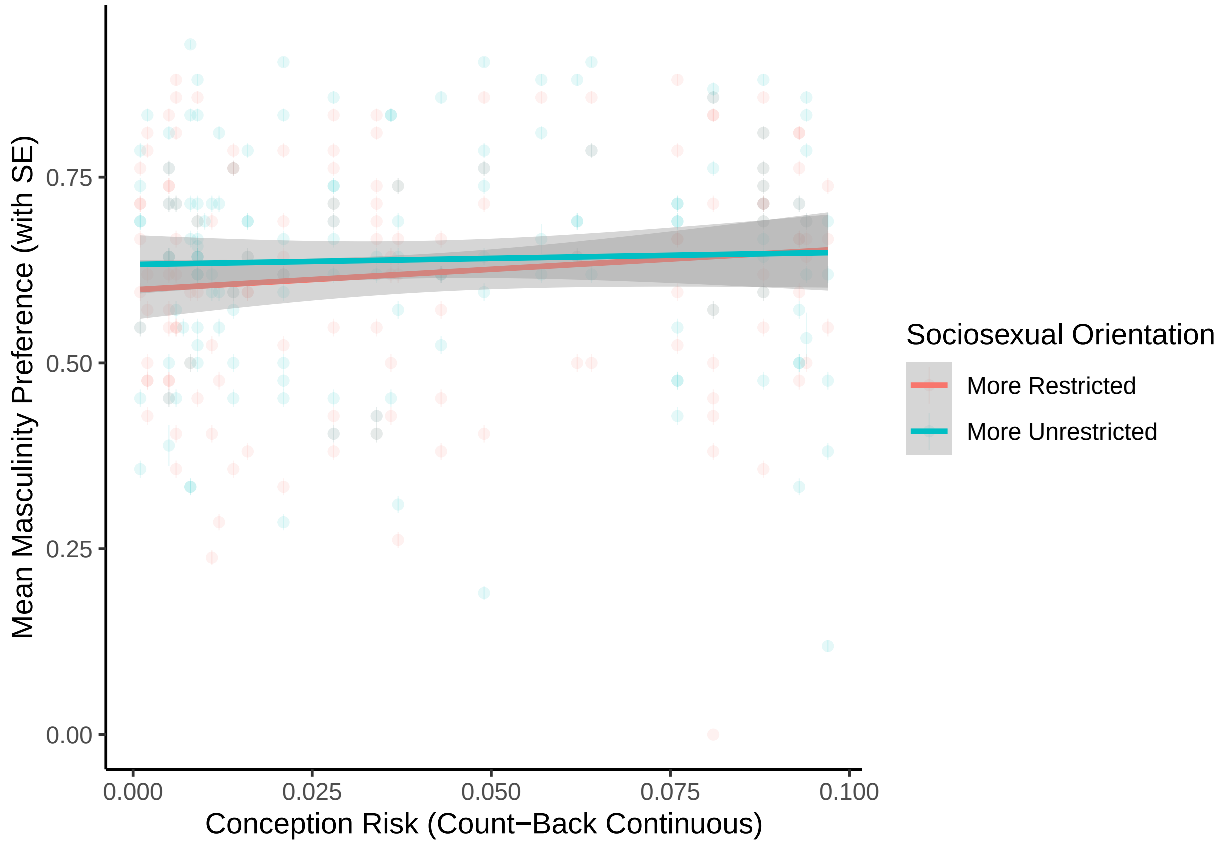


```
ggsave(filename = "fig3.png")
```

Count-Back Dichotomous

Linear Mixed Effects Model

```
if(file.exists("model.cb_dich.Rdata")){
  load("model.cb_dich.Rdata")
} else {
  model.cb_dich <- glmer(response ~ z.soi*e.cr_cb.dich +
                         (1 | ResponseId) +
                         (1 + z.soi*e.cr_cb.dich || faceId),data = analysis.data,family = "binomial")
  save(model.cb_dich,file = "model.cb_dich.Rdata")
}

summary(model.cb_dich)
```

```
## Generalized linear mixed model fit by maximum likelihood (Laplace
##   Approximation) [glmerMod]
##  Family: binomial  ( logit )
## Formula: response ~ z.soi * e.cr_cb.dich + (1 | ResponseId) + (1 + z.soi *
##     e.cr_cb.dich || faceId)
##    Data: analysis.data
##
##      AIC      BIC   logLik deviance df.resid
##  14533.4  14600.3  -7257.7  14515.4    12470
##
```

```
## Scaled residuals:
##     Min      1Q  Median      3Q     Max
## -4.2347 -0.8580  0.4176  0.6858  6.8423
##
## Random effects:
##  Groups     Name                Variance  Std.Dev.
##  ResponseId (Intercept)         5.130e-01 7.162e-01
##  faceId     (Intercept)         7.309e-01 8.549e-01
##  faceId.1   z.soi               1.187e-10 1.090e-05
##  faceId.2   e.cr_cb.dich        1.032e-02 1.016e-01
##  faceId.3   z.soi:e.cr_cb.dich 4.500e-10 2.121e-05
## Number of obs: 12479, groups:  ResponseId, 300; faceId, 42
##
## Fixed effects:
##                     Estimate Std. Error z value Pr(>|z|)
## (Intercept)         0.665241   0.141724   4.694 2.68e-06 ***
## z.soi               0.071391   0.052336   1.364    0.173
## e.cr_cb.dich       -0.001554   0.104104  -0.015    0.988
## z.soi:e.cr_cb.dich -0.051094   0.104665  -0.488    0.625
## ---
## Signif. codes:  0 '***' 0.001 '**' 0.01 '*' 0.05 '.' 0.1 ' ' 1
##
## Correlation of Fixed Effects:
##             (Intr) z.soi  e.cr_.
## z.soi       -0.001
## e.cr_cb.dch  0.156 -0.015
## z.s:.cr_cb. -0.005  0.513 -0.004
## optimizer (Nelder_Mead) convergence code: 0 (OK)
## boundary (singular) fit: see ?isSingular
```

Plot Data

```
plot.data <- analysis.data %>%
  mutate(soi_group = factor(ifelse(soi >= median(soi,na.rm = TRUE),"More Unrestricted","More Restricted
         cr_cb.dich = factor(cr_cb.dich,levels = c("Low","High"))) %>%
  group_by(ResponseId,cr_cb.dich,soi_group) %>%
  summarise(
    mean.response = mean(response,na.rm = TRUE),
    se.response = sd(response,na.rm = TRUE)/n()
  ) %>%
  filter(!is.na(mean.response)) %>%
  filter(!is.na(cr_cb.dich)) %>%
  filter(!is.na(soi_group))

ggplot(plot.data,aes(x = soi_group,y = mean.response,fill = cr_cb.dich)) +
  geom_half_violin(data = filter(plot.data,cr_cb.dich == "Low"),side = "l") +
  geom_half_violin(data = filter(plot.data,cr_cb.dich == "High"),side = "r") +
  stat_summary(data = filter(plot.data,cr_cb.dich == "Low"),position = position_nudge(x = -.15)) +
  stat_summary(data = filter(plot.data,cr_cb.dich == "High"),position = position_nudge(x = .15)) +
  theme_classic() +
  xlab("Sociosexual Orientation") +
  ylab("Mean Masculinity Preference (with SE)") +
  labs(fill = "Conception Risk (Count-Back Dichotomous)") +
  theme(legend.position = "bottom")
```

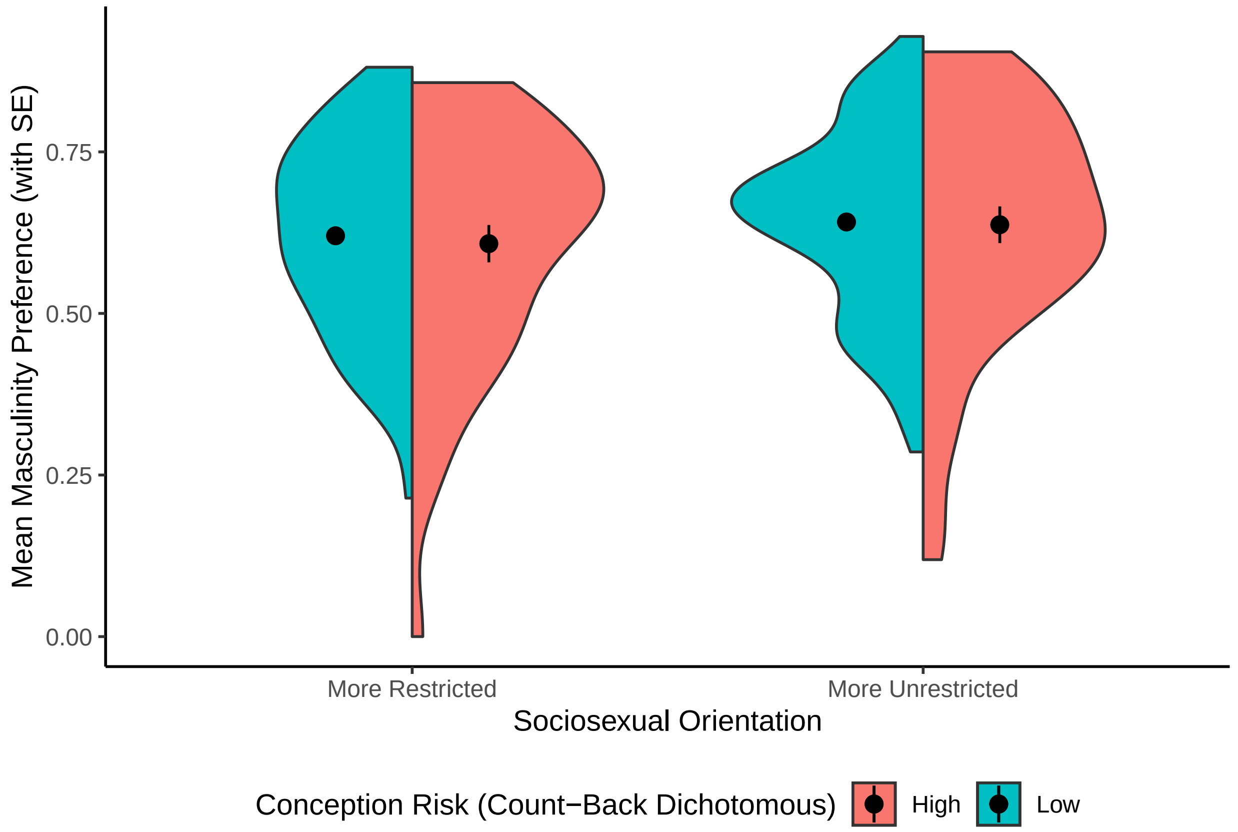


```
ggsave(filename = "fig4.png")
```
